# A Review on Chitosan’s Uses as Biomaterial: Tissue Engineering, Drug Delivery Systems and Cancer Treatment

**DOI:** 10.3390/ma13214995

**Published:** 2020-11-06

**Authors:** Rayssa de Sousa Victor, Adillys Marcelo da Cunha Santos, Bianca Viana de Sousa, Gelmires de Araújo Neves, Lisiane Navarro de Lima Santana, Romualdo Rodrigues Menezes

**Affiliations:** 1 Graduate Program in Materials Science and Engineering, Laboratory of Materials Technology (LTM), Federal University of Campina Grande, Campina Grande 58429-900, Brazil; 2 Laboratory of Materials Technology (LTM), Department of Materials Engineering, Federal University of Campina Grande, Campina Grande 58429-900, Brazil; gelmires.neves@ufcg.edu.br (G.d.A.N.); lisiane.navarro@ufcg.edu.br (L.N.d.L.S.); romualdo.menezes@ufcg.edu.br (R.R.M.); 3 Center for Science and Technology in Energy and Sustainability (CETENS), Federal University of Recôncavo da Bahia (UFRB), Feira de Santana 44042-280, Brazil; adillys.santos@ufrb.edu.br; 4 Department of Chemical Engineering, Federal University of Campina Grande, Campina Grande 58429-900, Brazil; bianca@deq.ufcg.edu.br

**Keywords:** chitosan, biopolymer, tissue engineering, drug delivery system, cancer treatment

## Abstract

Chitosan, derived from chitin, is a biopolymer consisting of arbitrarily distributed β-(1-4)-linked D-glucosamine and N-acetyl-D-glucosamine that exhibits outstanding properties— biocompatibility, biodegradability, non-toxicity, antibacterial activity, the capacity to form films, and chelating of metal ions. Most of these peculiar properties are attributed to the presence of free protonable amino groups along the chitosan backbone, which also gives it solubility in acidic conditions. Moreover, this biopolymer can also be physically modified, thereby presenting a variety of forms to be developed. Consequently, this polysaccharide is used in various fields, such as tissue engineering, drug delivery systems, and cancer treatment. In this sense, this review aims to gather the state-of-the-art concerning this polysaccharide when used as a biomaterial, providing information about its characteristics, chemical modifications, and applications. We present the most relevant and new information about this polysaccharide-based biomaterial’s applications in distinct fields and also the ability of chitosan and its various derivatives to selectively permeate through the cancer cell membranes and exhibit anticancer activity, and the possibility of adding several therapeutic metal ions as a strategy to improve the therapeutic potential of this polymer.

## 1. Introduction

The current interest in green technology has led to studies on biopolymers and bio-based polymers. They have excellent physical and biological properties and are biodegradable, unlike their equivalent synthetic polymers [1]. Chitosan is a good example, as it can be derived from partial deacetylation of chitin, a natural polymer found in various organisms [2].

Although chitosan’s discovery dates back to the XIX century, it came into the scientific “spotlight” three decades ago. Its importance to biomedical and technological applications has grown tremendously due to its functional character [3].

Chitosan exhibits several interesting biological and physicochemical properties, such as pH sensitivity, low-immunogenicity, biocompatibility, low toxicity, biodegradability, and bacteriostatic effects. Accordingly, chitosan and its derivatives are used in tissue engineering, wound healing, drug delivery, and anticancer materials [4].

Chitosan alone or in combination with other polymers or bioceramics has been used recently for tissue engineering, drug delivery systems, and cancer treatment applications [5]. In the form of a scaffold, chitosan acts as a physical support for cells and tissues, contributing to the structuring process of tissue, allowing countless cells to be seeded, migrate, and proliferate [6,7]. Moreover, it increases osteoblast activity, mineralization, collagen production, tissue regeneration, and hemostatic action, making chitosan one of the most prominent materials for tissue engineering applications.

Chitosan-based materials have been extensively studied in oral administration for drug delivery and in systems for topical delivery, colon-targeted drug delivery, gene delivery, and even carcinoma therapy due to their biocompatibility; abilities to serve as reaction sites with other bioactive compounds, and protect unstable drug molecules from strong gastric acids and blood flow responses; ability to adhere to mucosal tissues to improve the absorption of specific drugs; and convenience when combining them with anionic biomacromolecules such as DNA by electrostatic action [8]. Chitosan is soluble in dilute acidic solutions since it has primary amino groups (pKa = 6.3) [9], which makes CS a potential material for the oral administration of anticancer drugs.

Chitosan has inhibitory effects on tumor cell growth, tumor-induced angiogenesis, and tumor metastasis, thereby showing good anticancer activity. It is also possible to obtain a synergistic cytotoxic effect in cancer cells achieved by the co-administration of chitosan-based systems associated with therapeutic metal ions (TMIs), and drugs used in chemotherapy, improving the therapeutic potential and effectively reducing the tumor cell proliferation and contributing to accelerating the shrinkage and disappearance of tumors. Additionally, chitosan can induce innate immune responses by directly activating dendritic cells (DCs) (that can start responses of the innate immune system) and facilitating the cross-talk among DCs and natural killer (NK cells). Chitosan can promote the survival and improve the effector functions of human NK cells, implying in an in vivo antitumor activity [10,11].

Chitosan and its derivatives have had high-level performances in tissue engineering, drug delivery systems, and cancer treatment areas; there has been a significant increase in the number of studies concerning such things in the last ten years. However, there is a lack of systematic reviews on the recent literature in these potential chitosan application segments, which motivated the development of this review article.

## 2. Chitin and Chitosan

Chitin, the second most abundant organic compound found in nature [12,13], is a semi-crystalline polysaccharide consisting of β-(1→4)-2-amino-2-deoxy-D-glucose and β-(1→4)-2-acetamide-2-deoxy-D-glucose monomeric units. It is a biological polymer consisting of glucosamine and N-acetylglucosamine monomers linked to β-(1→4)-glycosidic bonds. The average molar weight of native chitin is generally greater than 10^6^ Daltons [14,15,16,17,18], and this material has interesting properties, such as biodegradability, antibacterial activity, non-toxicity, and gel-forming properties. Chitin is derived from a wide variety of sources, including shrimps, crabs, and crawfish (the principal ones), but also naturally exists in a few species of insects, fungi, and yeast [19,20].

**Figure 1:**
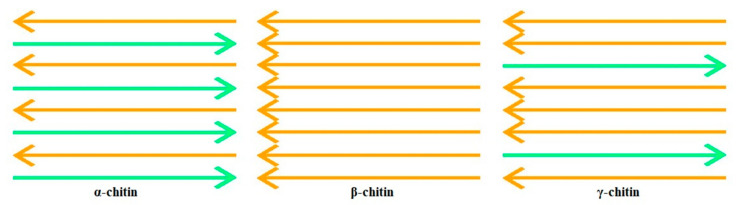
Schematic representation of the three polymorphic forms of chitin.

There are two main polymeric forms in which chitin exists—α-chitin and β-chitin, which are respectively arranged according to the monoclinic and orthorhombic cells. Compared to α-chitin, β-chitin, which can be extracted from squid pens, shows higher solubility, reactivity, and swelling, but α-chitin is preferably used in research laboratories and industries due to its greater dissemination in biomass, especially as an essential component of crustacean shells [21,22]. There is also a third form of allomorph, γ-chitin, which resembles a combination of α and β forms, instead of a different allomorph. When comparing the β and γ forms, the structure of α-chitin was further investigated, since it is the most common polymorphic form. As shown in Figure 1, α-chitin has a compacted orthorhombic cell, composed of alternating leaves of parallel and antiparallel chains; in β-chitin the adjacent leaves on the c-axis extension have the same direction; and γ-chitin, in turn, presents an alternating arrangement of polymer chains, where each third leaf has the contrary direction to the antecedent two leaves [13,23,24].

**Figure 2:**
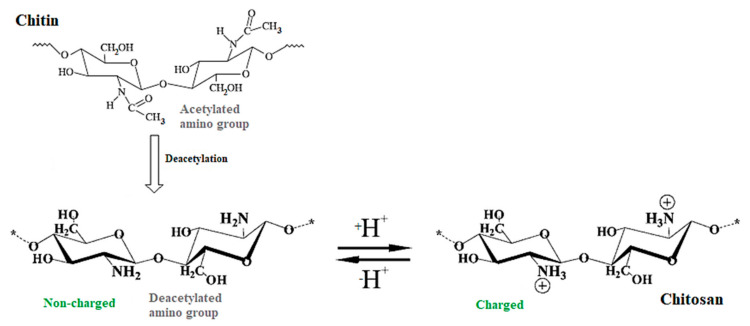
Chemical structures of chitin and chitosan.

Chitin possesses a linear chain of acetylglucosamine groups. The removal of enough acetyl groups through the deacetylation process gives rise to chitosan, which has a high concentration of reactive amino groups (−NH_2_) and presents solubility in most dilute acid solutions. This process occurs by enzymatic hydrolysis in the company of specific enzymes or chemical hydrolysis under drastic alkaline conditions [2,25]. Figure 2 illustrates the chemical structures of chitin and chitosan.

The degree of deacetylation (DD) varies with the hydrolysis conditions and chitin sources. This impacts chitosan’s chemical and physical properties, biodegradability, and immunological activity, and determines its appropriate applications [26]. The raw material and preparation methods influence the DD of chitosan, which spans from 56% to 99%, with a midpoint of 80%. Additionally, the term chitosan is usually used in the literature when the material has a DD above 70% [27,28]. Thus, chitin can be differentiated from chitosan according to the DD, since it indicates the percentage of free amino groups present in the polysaccharides [29].

Compared to chitin, chitosan presents two advantages: there is no need to use toxic solvents for dissolution; it dissolves in dilute weak acid solutions, such as acetic acid, due to the protonation of its amino groups. These groups are active sites in various chemical reactions, allowing numerous chemical modifications and enhancing solubility, biocompatibility, active targeting, etc. [30].

Chitosan’s characteristics make this material very interesting for biomedical applications [4]. The biodegradability of chitosan results from the presence of subsequent glycosidic bonds that can be broken. The in vivo degradation of chitosan occurs by several proteases, especially lysozyme. It is worth noting that, so far, eight human chitinases (a family of proteins widely expressed in the artery, in prokaryotes, and in eukaryotes) were recognized, three of them showing enzymatic activity in chitosan. This polymer biodegradation implies the generation of changeable length non-toxic oligosaccharides that can be excreted or integrated into the metabolic pathways. Additionally, this polysaccharide’s degradation ratio is associated with the molecular mass of chitosan, the distribution of N-acetyl D-glucosamine, and mainly, its DD [2].

Chitosan exhibits several interesting biological properties: antimicrobial, antibacterial, antifungal, antitumor, and hemostatic activities; clotting time reduction; mucoadhesion; analgesic; healing acceleration; treatment of osteoarthritis; a hypocholesterolemic effect; and a hypolipidemic effect [2,31,32,33]. To describe the antibacterial and antifungal activities of chitosan, two principal mechanisms have been referenced in the literature that can be combined to describe these activities [33,34,35,36,37]. The first proposed mechanism relates to the interaction of negatively charged groups at the cell surface with positively charged chitosan, changing its permeability, which would avoid the entry of essential materials into the cells or the pouring of fundamental solutes out of it. In turn, the second mechanism uses the linkages of chitosan’s protonated amino groups with the cell DNA, leading to the blocking of microbial RNA synthesis. This natural antibacterial and/or antifungal characteristics of chitosan and its derivatives justify its use in commercial disinfectants, where it impedes the development of an ample variety of fungi and bacteria. It has a broad spectrum of activity, in addition to less toxicity to mammalian cells when compared to other types of disinfectants [34].

The hemostatic activity of chitosan is noteworthy; it is more effective than that presented by chitin, and may be related to the positive charges present in the chitosan backbone, allowing interaction with negatively charged red blood cell membranes. Thus, chitosan’s hemostatic property implicates in the agglutination of red blood cells, inducing the formation of clots in the privation of platelets or coagulation factors, which can be helpful for patients therapeutically anticoagulated or with coagulopathies [38,39,40]. There may also be a relation among the positive charges in the chitosan skeleton and the negative part of the cell membrane, resulting in the reorganization and opening of the tight junction proteins, which explains the improvement in this polysaccharide permeation property [41]. Chitosan also presents analgesic effects that can be explained by its polycationic nature. The proton ions liberated in the inflammatory area result in the protonation of the amino groups present in the D-glucosamine residues, implying in an analgesic effect [42].

The increased interest in chitosan’s biomedical applications has generated opportunities for the production of specialized biomaterials, especially with new chemical and physical modifications, which have promoted new biological activities for specific purposes. These strategies have also involved combining chitosan with other polymers and inorganic materials to produce composite materials [43].

The majority of these chitosan applications are related to amino groups’ existence along with the residues of D-glucosamine that present polyelectrolyte and chelate properties [18,44,45]. Chitosan mucoadhesion, for example, can be explained by the interaction of these positively charged groups with the negatively charged residues (sialic acid) in mucin. It is also worth pointing to the relation of mucoadhesion with the chitosan degree of deacetylation: if chitosan DD increases, it enhances the number of positive charges and the permeation ability, which implies better mucoadhesive properties [46].

The use of chitosan faces two principal obstacles. One results from the fact that commercial products display variance in both DD (70–90%) [47] and average molecular weight (1.0 × 10^5^ to 1.2 × 10^6^ Daltons or 100 kDa to 1200 kDa), according to their origin (shrimp, crab, lobster shells, etc.). Additionally, chitosan is a weak polybase soluble in water in a limited range of pH (pH < 6.1) [45]; this low solubility limits its applications and necessitates chemical modification for any advancement in use. It involves introducing the functional groups without altering this polymer’s fundamental skeleton, maintaining the primary physicochemical and biochemical properties, and leading to new or enhanced properties [48].

### Chitosan’s Chemical Modifications

The conversion of chitosan into its oligosaccharides is well investigated in biological fields, since they require specific biological activities, such as antitumor activity, immunostimulatory effects, improvement of the protective effects averse to infection by some mice pathogens, antifungal and antimicrobial activities, radical elimination activity, and the anticoagulant activity of chitosan polysulfate derivatives reported by some researchers [38,49].

The chemical modification by replacing chitosan hydroxyl and amino groups with sulfate can cause a structural heterogeneity in the polymeric chain (structures randomly distributed), disclosing adequate characteristics for biological applications [50]. Sulfated chitosan and their derivatives with N and/or O-sulfate groups, and even combined with other substituents, have been extensively tested as potential heparinoids, and exhibited anticoagulant, antitumor, antisclerotic, and antiviral activities [51,52,53,54,55]. Studies have been showing a high anticoagulant potency when compared to the standard therapeutic heparin [38].

Vongchan and co-workers [50] modified chitosan from marine crab shells (DD = 0.89%) using chlorosulfonic acid/dimethylformamide under semi-heterogeneous conditions. The authors reported that the resulting chitosan polysulfate exhibited high solubility in common organic solvents and dissolved well in water. Additionally, chitosan polysulfate preparations revealed strong anticoagulant activities, direct inhibition of thrombin activity, and indirect inhibition the activity of factor Xa through antithrombin III activity—the same mechanism of anticoagulant activity observed in heparin. Park and co-workers [38] studied the anticoagulant properties of heterochitosans and chitosan oligosaccharide sulfates (COS) with different DD and molecular weights. Anticoagulant tests using normal human plasma revealed that the increases in the concentrations of heterochitosan and its COS sulfates led to distended activated partial thromboplastin and thrombin time. Additionally, the chitosan sulfates with a degree of deacetylation of 90% presented the greatest anticoagulant activity.

Various chemical derivatives of chitosan are reported in the literature [56]. Some possible reactions comprise the -NH_2_ group at the C-2 position; quaternization and the reductive amination with aldehydes are the most common and easy reactions. Additionally, the non-specific reactions of -OH groups in C-3 and C-6 positions are possible (mainly etherification and esterification) [57]. Water-soluble chitosan can be acquired by degradation or through chemical modification, such as carboxymethylation, a reaction that can occur at hydroxyl groups (forming O-carboxymethyl chitosan (O-CMC)), amino groups (to obtain N- carboxymethyl chitosan (N-CMC)), or both (producing N, O-carboxymethyl chitosan (N, O-CMC)). Carboxymethyl chitosan (CMC) has several biomedical applications, such as drug/gene delivery, wound healing, tissue engineering, and bioimaging [58].

The amphoteric polyelectrolyte *O-N*-carboxymethyl chitosan (CM-chitosan) achieved beneath controlled reaction conditions with sodium monochloroacetate is one of the most investigated derivatives of chitosan, owing to its unique biological and physicochemical properties and outstanding biocompatibility. It has substantial potential in numerous biomedical applications, including bacteriostatic agents, dressings, blood anticoagulants, and artificial bones and skin [59,60,61,62,63]. The physicochemical and biophysical properties result in amino and carboxyl groups’ presence in chitosan macromolecules—that being interesting for pharmaceutical applications, particularly for controlled drug delivery systems [64].

N-carboxymethylation of chitosan is carried out by forming the Schiff base from the free chitosan amino group with a keto group or aldehyde and sequent reduction with cyanoborohydride or sodium borohydride. This results in an amino group regioselective carboxymethylation, such that the reaction product is a well-defined derivative (carboxyalkylated derivatives) used as fungistatic agents and biomedical materials [65,66,67,68,69,70].

The amphiprotic ether derivative of chitosan (CS), O-carboxymethyl chitosan (O-CMC), contains the carboxyl (−COOH) and amino (−NH_2_) groups in the macromolecule, giving rise to unique physical-chemical and biophysical properties, and consequently, causing substantial attention to be received, especially for pharmaceutical applications in controlled or sustained drug delivery systems owing to its non-toxicity, biodegradability, biocompatibility, and antibacterial and antifungal activities [64,71].

The use of distinct acylating agents, such as cyclic anhydrides, aliphatic carboxylic acid chlorides, and cyclic esters, generates a diversity of chitosan acylation, not regioselective reactions. When preparing *N,O*-acylated chitosans with acyl chlorides in methanesulfonic acid [72,73], the derivatives of reactions with 4-chlorobutyl and decanoyl chlorides exhibited improved fungicidal activity when compared to chitosan [74].

## 3. Chitosan in Tissue Engineering

**Figure 3:**
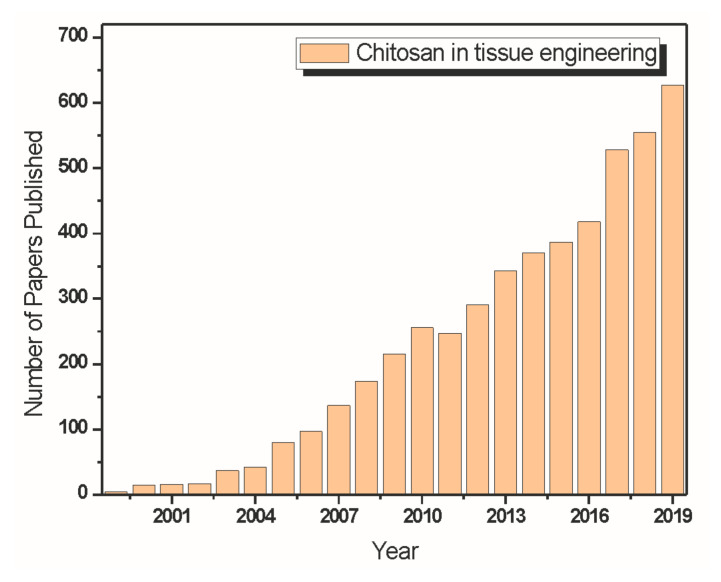
Literature data extracted from the Web of Science Core Collection about papers that address chitosan (CS) applied in the field of tissue engineering.

Chitosan has attracted attention in tissue engineering and regenerative medicine over the last few years. It has favorable properties, such as hydrophilicity, biocompatibility, biodegradability, and antimicrobial activities. Research on the development of scaffolds has emerged due to its functional character [75,76,77,78]. Figure 3 shows a graph made from statistics extracted from data from the Web of Science Core Collection about the number of papers published addressing chitosan applied in the field of tissue engineering over the last years.

The ease with which CS can be developed in distinct forms, such as gels, micro and nanoparticles, nanofibers, and scaffolds, is advantageous. Additionally, chitosan has an excellent ability to form porous structures [5] for use in tissue regeneration and cell transplantation. Various compositions were used to produce a scaffold with a pore size and mechanical properties desirable for several tissue engineering areas [79].

Chitosan porous scaffolds can be obtained by freeze-drying of CS solution or by processes such as an “internal bubbling process (IBP),” with the use of porogenic materials [80]. The highly porous structures with interconnected pores can improve in vitro and in vivo cell proliferation [81,82,83,84,85,86,87,88,89]. It is worth noting that, by modifying the freezing rate and the ice crystal size, the average pore size of this material can be controlled [90].

Another critical property of chitosan scaffolds is the intrinsic antibacterial activity. Studies revealed that the antibacterial activity of chitosan and chitosan-based systems is comparable to well-known antibiotics responses; additionally, CS’s antibacterial character is concentration-dependent. A level of activity similar to those of amikacin (30 mg) and erythromycin (15 mg) antibiotics was found, along with even more potency against bacteria than rifampicin (5 mg), penicillin (10 mg), and trimethoprim (25 mg). Thus, chitosan offers an excellent possible option for tissue engineering applications due to its facile ability to produce porous scaffolds and antibacterial activity [18,91,92].

### 3.1. Bone Tissue Engineering

Chitosan-based scaffolds have been extensively utilized in bone tissue engineering due to their high cell adhesion and proliferation [93,94]. This behavior is associated with the scaffold’s physical characteristics and electrostatic interactions (arising from the cationic nature of chitosan) [95] with many compounds, such as cytokines/growth factors. These compounds are responsible for improving cell colonization [90].

Hybrid CS-based scaffolds have been developed by a combination of CS with nanoceramics, such as hydroxyapatite (HAp), silicon dioxide (SiO_2_), titanium dioxide (TiO_2_), bioactive glass-ceramic (BGC), and zirconium oxide (ZrO_2_) [95]. Once implanted, chitosan induces a minimum foreign body reaction, furthering cell adhesion, proliferation, and differentiation. These hybrids combine each material’s desirable characteristics, taking advantage of the organic part’s flexibility and adequate molding capacity, and the inorganic part’s properties, such as thermal stability and chemical resistance [96]. Besides, as evidenced by a mesenchymal stem cell (MSC) culture in a SiO_2_–gelatin hybrid [97], SiO_2_–collagen composites [98], and an osteosarcoma cell line SaOs-2 on a poly(γ-glutamic acid)/SiO_2_ hybrid [99], the silica hybrid materials are not cytotoxic and favor cell attachment.

Hybrid nanofibers made of chitosan have been used to produce scaffolds. These hybrid nanofibers incorporated the benefits of different materials: they are cytocompatible, promoting the attachment and proliferation of cells, such as osteoblast-like 7F2-cells; and new adjustable properties can be incorporated when generating bioactive scaffolds for repairing of bone Toskas, et al. [100].

The use of calcium phosphates (CaP), such as hydroxyapatite (HA) and β-TCP, has been widely investigated in the production of chitosan–CaP hybrid scaffolds. However, the inorganic HA scaffolds’ ideal morphology and porosity present unsatisfactory mechanical properties [101]. The properties expected for chitosan/calcium phosphate scaffolds are biocompatibility, biodegradation, osteoconductivity, antibacterial effects, osteoinduction, angiogenesis regulation, and mechanical strength [102].

Hydroxyapatite (HA)/gelatin–CS core-shell nanofiber composite scaffolds were developed by coaxial electrospinning in the work of Chen et al. [103]. Chitosan and gelatin are located inside the core-shell nanofibers and considerably favor cell adhesion and proliferation, which is further increased by the presence of HA deposited on the surfaces of nanofibers. Gelatin–CS core-shell structured nanofibers enhanced HA’s mineralization efficiency and formed a homogeneous HA deposit when compared with CS, gelatin, and CS-gelatin nanofibers. The results evidence that HA deposited on the gelatin–CS core-shell nanofibers could further enhance osteoblast cell proliferation when the human osteoblast-like cell line (MG-63) is cultured on this material.

Oliveira, et al. [104] developed scaffolds of chitosan/hydroxyapatite and gelatin. It was possible to observe the particle aggregation method that successfully formed a three-dimensional structure (scaffolds). By FTIR, hydroxyapatite was observed in different concentrations, identified by changes in the spectra obtained. As the amount of hydroxyapatite increased, there was a greater tendency to approach the curve with similar behavior to this isolated raw material. Additionally, through the mechanical characterization, it was possible to observe a pseudoplastic behavior in the curves obtained due to the polymeric fraction’s influence in the scaffold. The authors related that the hydroxyapatite acts as reinforcement until the concentration of 3%, and with the addition of 5%, the hydroxyapatite then decreased the mechanical strength of the scaffolds.

Chitosan-4-thio-butylamidine (CS-TBA), a water-soluble thiolate chitosan conjugate, was synthesized and utilized to prepare a CS-TBA/hydroxyapatite (HA)/disodium beta-glycerophosphate (-GP) thermosensitive hydrogel in the work of Liu et al. [105]. In vitro studies on the release behavior of cysteine terminated peptide 24 (P24) containing residues 73–92 of the BMP-2 knuckle epitope from CS-TBA based hydrogel showed a lower release rate and the maintenance of the release of this peptide for a longer time when compared to the unmodified chitosan system (CS/HA/-GP). This can be attributed to the thiol groups’ reaction in the CS-TBA with the thiol groups in P24. The bioactivity of P24 was maintained during the release process, which may, in turn, have been associated with degradation of the chitosan network, since the covalent bound P24 was only released when the hydrogel broke down. The results indicate the potential for using this thermosensitive hydrogel as injectable support for bone tissue engineering.

Microporous methacrylated glycol-chitosan-montmorillonite nanocomposite hydrogel was developed in the work of Cui et al. [106]. The hydrogels formed an interconnected microporous network and promoted the proliferation, attachment, and differentiation of encapsulated mesenchymal stem cells in vitro. Additionally, CS-montmorillonite hydrogels were able to recruit native cells and promote calvarial healing without the delivery of additional therapeutic agents or stem cells, indicating a potential for tissue engineering.

Gritsch, et al. [107] studied a Cu–CS derivative and a Sr-substituted HA combined in composite scaffolds. The results showed a great potential for combining CS and HA, together with Cu and Sr, to develop new scaffolds. While strontium is uniformly released to assist the regeneration process for an extensive time, copper has an explosive release from chitosan, enabling possible bacterial colonization to be quickly stopped.

Jahan et al. [108] developed apatite-CS scaffolds through a rapid purine-crosslinking reaction as a potential injectable construct for bone tissue engineering. Morphological analyses revealed an interconnected, highly porous structure. The scaffold presented an osteogenic-like microenvironment adequate for biomineralization. MC3T3 preosteoblast cell differentiation and subsequent biomineralization, revealed by ALP enzymatic activities over seven days of culture and in vitro studies, showed good morphology and cell adhesion.

Genipin (GP)-crosslinked and fucoidan (FD)-adsorbed nano-hydroxyapatite/hydroxypropyl chitosan composite scaffolds were developed in the work of Lu et al. [109]. Initially, the synthesized n-HA was incorporated into the HPCS solution, followed by crosslinking the n-HA/HPCS blends with GP and FD’s adsorption to the n-HA/HPCS composite scaffolds. The genipin crosslinking of HPCS and the inclusion of n-HA in the scaffolds reduced the hydrophilic property, controlled the porous architecture, improved mechanical strength, and decreased degradation. Additionally, the incorporation of n-HA caused an open structure with interconnected pores and a rough morphology, and in addition to the adsorption of fucoidan, increased ALP activity in 7F2 osteoblast cells and promoted their mineralization.

CS/carboxymethyl cellulose reinforced with multiphasic calcium phosphate whisker-like fibers was fabricated by the freeze-drying method in the work of Matinfar et al. [110]. The composite scaffolds exhibited a high porosity microstructure with interconnected pores, and scaffolds presented improved in vitro proliferation, attachment, and mineralization of MG63 cells, as evidenced by MTT assay, DAPI staining, SEM, and Alizarin red staining. Additionally, the addition of CMC to CS led to a significant improvement in the mechanical properties, and the composite scaffolds of CS and CMC reinforced with 50 wt.% triphasic fibers were superior in terms of mechanical and biological properties, reinforcing the scaffolds and resulting in higher cell viability.

Chitosan scaffolds reinforced with hydroxyapatite (HA) nanofiber were fabricated by the freeze-drying method in the work of Nezafati et al. [111] using glycidyloxypropyl-trimethoxysilane (GPTMS) as a crosslinking agent. An increase in the porosity percentage and a decrease in the average pore size when GPTMS was added to the chitosan structure were observed. When n-HAs were added, there was an increase in the porosity percentage and pores was more irregular in shape than pure chitosan and chitosan scaffolds. After 14 days of soaking in the SBF solution, proper biomineralization behavior and improved viability of MG-63 osteoblastic cells concerning the chitosan scaffold for up to 72 h of cell culture were observed, demonstrating potential applicability of the CGH25 scaffold as an appropriate alternative for bone substitutes.

Chitosan can also be combined with alginate (Alg) to generate a polyelectrolytic complex, leading to mutual precipitation and improved mechanical strength [112,113]. The alginate helps in cell regeneration, while CS gives the supports structure [114]. The strong connection between them is also because Alg is a hydrophilic polysaccharide that further lowers protein adsorption. At the same time, CS is a less hydrophilic polysaccharide that promotes elevated protein adsorption [115,116], giving rise to CS–Alg composites largely used for drug and protein delivery, tendon and ligament tissue engineering, wound healing, and intervertebral tissue engineering [117].

Scaffold structures composed of CS–Alg and CS–Alg–fucoidan were generated by Venkatesan, Bhatnagar and Kim [117]. Good cytocompatibility increased alkaline phosphatase secretion, and enhanced cell proliferation was evidenced in the CS–Alg–fucoidan scaffold by in vitro studies using the MG-63 cell line. Additionally, protein adsorption and mineralization were ameliorated in comparison with the CS–Alg scaffold.

Chitosan/alginate/hydroxyapatite/nanocrystalline cellulose scaffolds were developed by the freeze-drying method, followed by dicationic crosslinking using CaCl_2_. All scaffolds possessed highly porous 3D structures with good interconnectivity and rough edges, facilitating cell binding and proliferation. Osteoblast MG63 cells were cultured on the 3D porous scaffold, and results indicated the proliferation and enrichment of osteoblast cells [118].

Scaffolds made of chitosan combined with poly-3-hydroxybutyrate (PHB) had favorable physical and mechanical properties with no observed undesirable chronic inflammatory response, even one year after implantation. This combination improved the low degradation rate of PHB, one of the most important limitations of its application in tissue engineering. The mechanical properties of the material can be increased by applying ceramics as a reinforcement phase. The bioceramics like ZrO_2_ and Al_2_O_3_ are more effective than hydroxyapatite because of their high Young’s moduli and tensile strengths [119].

Toloue et al. [119] developed poly (3-hydroxybutyrate)-chitosan/alumina nanowire composite scaffolds by electrospinning. After 7 days of immersion in SBF, apatite deposited on the surface of the nanofibrous scaffolds. In vitro analysis showed high proliferation and viability of MG-63 cells and alkaline phosphatase secretion.

Saekhor et al. [120] developed a CS-based hydrogel by modifying CS with carboxymethyl chloride. The resultant carboxymethyl chitosan (CMCTS) was conjugated to α-cyclodextrin (α-CD) to produce CMCTSCD, which presents improved water solubility, and the hydrophobic cavities function as crosslinking points by forming an inclusion complex with poly(ethylene glycol) (PEG). The hydrogel had an interconnected porous structure suitable for transporting extracellular fluid, nutrients, and hormones to cells. The human osteogenic sarcoma cell line (SaOS-2) adhered and proliferated well on the hydrogel.

### 3.2. Cartilage Tissue Engineering

Tissue engineering techniques have been considered a favorable option for repairing articular cartilage [121,122]. Cell-based therapies are an alternative to promote the generation of similar tissues and promote this repair by selecting an appropriate cell source and a biomimetic matrix to host the cells [123,124,125,126].

Hydrogels can be used to partially mimic the stromatolytic structures and anisotropic compositions of the cartilage matrix, promoting their repair [127,128,129]. Thus, the widespread use of chitosan-based hydrogels stands out for the regeneration of cartilaginous tissue, since this polymer has a structure similar to glycosaminoglycan (GAG), a key component in the cartilage matrix, in addition to biocompatibility, biodegradability, bioadhesion, cell affinity, and intrinsic antibacterial, chondro-conductive, and chondro-integrative properties [96,127,130].

Chitosan hydrogels have the potential for cell-based tissue repair since they efficiently support chondrogenic activity, maintain the round morphology of chondrocytes, and preserve their ability to synthesize the cell-specific extracellular matrix, allowing expression of ECM cartilage proteins by chondrocytes. Chitosan administered by intra-articular injection promotes thicker epiphyseal cartilage in the tibial and femoral joints, with the proliferation of chondrocytes. These characteristics make this polymer a potential material for use in cartilage tissue engineering to modulate chondrocyte morphology, modulate differentiation, and stimulate chondrogenesis [96,131,132,133,134,135,136,137].

However, unmodified chitosan-based hydrogels are generally inefficient for cartilage repair due to their low strength and elasticity, fast in vivo degradation, and limited capacity for tissue adhesion, which can be attributed to weak interactions with tissues without the formation of mutually intertwined chains between the two contact interfaces [127,130,138,139,140,141]. In this context, the conjugated catechol groups in the chitosan backbone promote covalent bonds between oxidized catechol groups and amine or thiol groups present in the proteoglycan structure, providing additional adhesion strength to tissue surfaces [142,143,144].

Thermosensitive and injectable adhesive hydrogels of catechol-conjugated chitosan and thiol-terminated Pluronic tissue were developed in the work of Ryu et al. [145]. The hydrogels showed excellent mechanical properties, in vitro and in vivo stability, strong adhesiveness to soft tissues and mucous layers, and superior hemostatic properties. After injection, they were able to immediately form adhesive gels to the tissue. Additionally, they presented arrest bleeding properties, which allows the use of this material in the administration of injectable drugs, tissue engineering hydrogels, tissue adhesives, and antibleeding materials.

A composite hydrogel derived from N-succinyl-chitosan (S-CS), and aldehyde hyaluronic acid (A-HA) was prepared in the work of Tan et al. [146]. The encapsulation of bovine articular chondrocytes demonstrated the potential of the compound hydrogel as an injectable scaffold within the compound hydrogel matrix in vitro. The results showed that the hydrogel compound promoted cell survival. The cells maintained the regular spherical chondrocytic morphology, supporting cell adhesion and indicating its potential application in cartilage tissue engineering.

Water-soluble chitosan derivatives, chitosan-graft-glycolic acid (GA), and phloretic acid (PA) (CS-GA/PA) were designed in the work of Jin et al. [137] to produce biodegradable injectable chitosan hydrogels through enzymatic crosslinking with horseradish peroxidase (HRP) and H_2_O_2_. Good solubility of CS-GA/PA hydrogel was observed with a GA degree of substitution (DS) of 43 and PA degree of substitution of 10 (CS-GA43/PA10) (DS being defined as the number of substituted NH_2_ groups per 100 glucopyranose rings of chitosan) at pH values up to 10. These hydrogels can be readily degraded by lysozyme. In vitro culturing of chondrocytes in CS-GA43/PA10 hydrogels revealed that, after 2 weeks, the cells were viable and retained their round shape, indicating that the CS-GA/PA hydrogels constitute a promising artificial extracellular matrix for cartilage tissue engineering.

Anionic salts, such as sodium glycerophosphate (GP), are excellent when trying to produce chitosan/GP gel with thermosensitive characteristics that can deliver different cells while supporting its growth [140]. Crosslinking of these hydrogels with the use of hydroxyethyl cellulose improved gelation, and their use has been demonstrated in the repair of articular cartilage defects (AC), the encapsulation of intervertebral disc (IVD) cells, and the accumulation of a functional extracellular matrix that mimics that of the nucleus pulposus (NP) [147]. All the formulations of the in vitro cell-seeded chitosan hydrogels evidenced that most of the proteoglycan produced by encapsulated NP cells was retained in the gel instead of being released into the culture medium, which indicates that chitosan may be a suitable scaffold for cell-based supplementation to assist in the restoration of the NP function during the early stages of IVD degeneration.

An injectable and adhesive chitosan-based cell-delivery vehicle was developed in the work of Hoemann et al. [148]. The developed cytocompatible chitosan solution is space-filling and adheres to cartilage in situ, suggesting a potential for use as an arthroscopically injectable vehicle for cell-assisted cartilage repair. The chitosan gel was cultured in vitro, with and without chondrocytes, and injected subcutaneously in nude mice to form subcutaneous dorsal implants. The histochemical, biochemical, and mechanical properties of the resulting tissue constructions are comparable to those observed in vitro for primary chondrocytes cultured in 2% agarose. After being injected, the gel was retained for 1 day in vivo in a chondral defect of the full thickness of a rabbit, and for up to 1 week in osteochondral defects of rabbits, indicating the capacity of the gelling chitosan solution—while persisting in osteochondral defects for at least 1 week in vivo—supports the in vitro and in vivo accumulation of the cartilage matrix by primary chondrocytes.

A thermoresponsive chitosan-g-poly(N-isopropylacrylamide) (CS-g-PNIPAAm) copolymer was synthesized in the work of Mellati et al. [126] as a carrier of mesenchymal stem cells (MSCs) to provide support for their proliferation and differentiation. CS-g-PNIPAAm hydrogels were loaded with 3D microengineered cells with different microstrip widths to control cell alignment and elongation better to mimic the superficial zone of natural cartilage better. After 28 days of incubation in a chondrogenic medium, the MSCs encapsulated in the synthesized hydrogel showed 6 and 7 fold increases in glycosaminoglycans’ (GAGs) secretion and total collagen, according to the biochemical tests.

Hydroxybutyl CS (HBC)/oxidized chondroitin sulfate (OCS) hydrogels were fabricated in the work of Li et al. [149] by 3D bioprinting technique. The synergistic association of two biopolymers enabled good injectability of the composite hydrogel. The favorable biocompatibility allowed the 3D in vitro culturing of human adipose-derived mesenchymal stem cells (HAMSCs) with high viability. Additionally, HBC/OCS hydrogels provoked low inflammatory gene expression of macrophages in vitro, and inadequate inflammatory responses in vivo, inhibiting acute immune responses in 7 days.

Micro/nanocomposite hydrogels of photocrosslinkable maleilated chitosan (MCS)/methacrylated silk fibroin (MSF) were prepared in the work of Zhou et al. [150] by an MSF micro/nanoparticles dispersion in MCS aqueous solutions followed by the photopolymerization process. The hydrogel exhibited the compressive modulus of 0.32 ± 0.07 MPa when the MSF content was 0.1%, a value in the articular cartilage compressive modulus range. In vitro cytotoxic evaluation and cell culture evidenced the biocompatibility of the hydrogels with TGF-β1 with mouse articular chondrocytes and the capacity to support cell attachment well, indicating a great potential as tissue engineering scaffolds for cartilage repair.

Thiolated chitosan (CS-NAC) was synthesized in the presence of silk fibroin (SF) to produce dual network CS-NAC/SF hydrogels in the work of Liu et al. [151] for cartilage tissue engineering. In the CS-NAC/SF hydrogels, the CS-NAC component enhanced strength and stiffness, while the SF component contributes to the hydrogel elasticity. Additionally, CS-NAC/SF hydrogels revealed a highly porous structure with well-interconnected pores, which allows the culture and growth of chondrocytes while effectively maintaining their phenotype.

Polyhydroxybutyrate (PHB) was blended with chitosan (CS) using TFA as a co-solvent to electrospin the blend solution into fibrous scaffolds [152]. The presence of PHB increased the hydrophilicity and mass loss percentage of the scaffolds with the addition of CS, and kept the mechanical properties in an appropriate range for the cartilage tissue engineering application. Additionally, the chondrocytes attached well, spread, and slightly penetrated the polymer matrix of the fibers, being more adequate than the pure PHB scaffold, in which the cells attached to the fibers did not spread well [152].

Chitosan (CS)/poly(vinyl alcohol) (PVA)/graphene oxide (GO) composite nanofibers were fabricated by electrospinning in the work of Cao et al. [153]. The addition of GO increased the mechanical properties of the nanofibers. Cell proliferation assay was performed after day 14 to evaluate the biocompatibilities of nanofibers towards ATDC5 cells, and the results showed that the CS/PVA/GO (6% by weight) composition provides a suitable environment for the growth of ATDC5 cells.

Shamekhi et al. [154] developed graphene oxide (GO)-chitosan (CS) scaffolds for cartilage tissue engineering. Physical and mechanical properties were considerably improved with the rise in GO content. Additionally, the increase in the percentage of GO augmented the proliferation of human articular chondrocytes, especially in extended cultivation periods (14 days).

### 3.3. Neural Tissue Engineering

Peripheral nerve damage is a global clinical problem with a high incidence in today’s society that impairs quality of life for thousands of patients [155,156,157]. Chitosan has attracted significant attention among the various biomaterials proposed for the formation of nerve conduits due to its biocompatibility, biodegradability, low toxicity, and antibacterial effects—its potential for nerve regeneration was demonstrated in several in vitro and in vivo studies [158,159,160,161,162,163,164].

A freeze-dried chitosan gel sponge was examined in the work of Ishikawa et al. [163] as a scaffold for nerve regeneration in rats. A gap of 8 mm was made with the removal of a segment of the sciatic nerve, and a chitosan gel sponge sandwiched the distal and proximal stumps. Four days after the operation it was possible to observe the regenerating axons, and 14 days later, nerves extended the distal stump. Several macrophages seemed to phagocytize chitosan, implying in the formation of a dense cell layer. The regenerating axons did not touch the chitosan and extended across the space surrounded by this polymer stacked by macrophages. It was also observed that after 2 months of surgery, the regenerating nerves were well myelinated, and their diameters after 2 and 4 months of surgery were, on average, 2.45 and 2.75 mm, respectively. The results presented in this work indicate the possibility of using the chitosan gel sponge sandwich as a graft for peripheral nerve regeneration.

A laser-activated adhesive sheet based on chitosan, indocyanine green, acetic acid, and water was used to perform the in vivo anastomosis without suturing the rats’ tibial nerves. Strips were bonded to the sciatic nerves of rats and sheep’s intestines by laser activation with low fluence (50 J/cm^2^) to test the adhesive strength in vitro. Good adhesion to the tissue was observed with a tensile strength of 12.5 ± 2.6 kPa, which was suitable to maintain the in vivo continuity of the anastomosed nerves 3 days after surgery. Additionally, when compared to intact nerves, the number and morphology of myelinated axons were typical (ca. 96%) [165].

Wrobel et al. [157] analyzed the biocompatibility of Schwann cells (SCs) and bone-marrow-derived mesenchymal stromal cells (BMSCs) on chitosan films, and the results demonstrate a good cytocompatibility of the chitosan substrate. This allowed neurites to grow from dissociated sensory neurons, showing a high potential of this polymer for tissue-engineered nerve grafts.

Chitosan derived from crab tendons is promising for creating hollow tubular structures, useful in nerve regeneration. However, chitosan tubes have two problems that need to be solved: the low mechanical strength presented by the tubes for lateral pressure and the swelling of the tube walls that reduces the tubes’ internal space in vivo, limiting their effective use for nerve regeneration. To solve these problems, apatite was incorporated into the chitosan tubes to increase the mechanical strength of the tube walls in the work of Yamaguchi et al. [161]. The formation of apatite crystals on the walls of the chitosan tubes was identified by transmission electron microscopy, and a good alignment of the crystals’ c-axis was observed in parallel with the chitosan molecules, indicating that the growth of these crystals occurs from the nucleation sites of chitosan molecules, probably by forming complexes with amino groups of chitosan and calcium ions. Animal tests using male Sprague–Dawley (SD) rats showed that the chitosan tubes effectively induced nerve tissue regeneration, while being gradually degraded and absorbed in vivo.

The mechanical and biological properties of chitosan can be improved by mixing it with collagen or gel polymers, such as gelatin (Gel), which presents free carboxyl groups that can interact with the cationic groups of chitosan, resulting in the formation of a network by hydrogen bonding [166]. These chitosan–gelatin mixtures give rise to a structural scaffold for embryonic stem cells or bone marrow mesenchymal stem cells-based tissue engineering [167,168].

Chitosan (Cs)/gelatin (Gel) porous scaffolds containing hyaluronic acid (HA) and heparan sulfate (HS) were fabricated via freeze-drying in the work of Guan et al. [169]. Cs/Gel/HA/HS composite scaffolds presented very homogeneous and interconnected pores with porosity above 96% and a controllable degradation rate. Cell culture studies showed that the presence of HA and HS in the Cs/Gel/HA/HS scaffolds promoted relevant initial NS/PCs adhesion and supported the long term growth in a 3D environment, and compared to the Cs/scaffolds Gel, NS/PCs in the Cs/Gel/HA/HS also maintained multilinear differentiation potentials with increased neuronal differentiation.

Despite the effectiveness of chitosan and gelatin blends for nerve, skin, cartilage, bone, and muscle tissue engineering [169,170,171,172,173], they have limited applications in areas where electrical stimulation is required due to the low electrical conductivity presented by those polymers. In the context of neural tissue engineering, electrical stimulation can increase neuritis and axon regeneration in vitro and nerve regeneration in vivo. Polyaniline (PANI) is a biocompatible, organic conducting polymer that has been used to stimulate neural growth and regeneration in vitro and in vivo [174,175,176,177].

Conductive polyaniline/graphene (PAG) nanoparticles were incorporated into a chitosan–gelatin matrix in the work of Baniasadi et al. [177], aiming at the formation of porous conductive scaffolds for potential application in the repair of peripheral nerves. Increases in the scaffolds’ electrical and mechanical properties were observed with the incorporation of PAG, along with decreases in the porosity and the water retention capacity. Besides, compared to pure CS/gel scaffolds, conductive scaffolds exhibited relatively slower degradation and the largest number of attached Schwann cells, according to in vitro tests.

A biocompatible, self-gelling electroactive hydrogel based on a chitosan–aniline oligomer and agarose was synthesized in the work of Bagheri et al. [178] to recapitulate the niche of neural cells to achieve appropriate cell activity. The hydrogel’s conductivity was −10^−4^ S/cm, which could mimic the neural tissue. The aniline oligomer played a fundamental role in controlling the properties of the hydrogel, since it prolonged the release rate of the drug and rendered electroactivity to the hydrogel, increasing cellular proliferation.

Polycaprolactone (PCL)/chitosan (CS) blend scaffolds with different CS amounts were prepared by electrospinning in the work of Bolaina-Lorenzo et al. [179]. As the chitosan content increased, so did the scaffold hydrophilicity. Additionally, the average diameter of the nanofibers decreased. The scaffold’s non-cytotoxicity was verified from the cytotoxicity assays using fibroblasts, and the adherence of Schwann cells (SC) to the scaffolds was proven from biological tests. High cell proliferation was observed on days 1 and 3 for the scaffold containing 5 wt.% of CS.

Polypyrrole (PPy)-conjugated polymer was incorporated in the PCL/CS, by electrospinning to form a scaffold for nerve tissue engineering and neural applications due to its excellent electrical properties and sufficient biocompatibility. FTIR experiments identified the presence of chitosan in the fiber structure. It promoted a considerable increase in the scaffold’s hydrophilicity, as confirmed by a reduction of up to 66% in the contact angle. An increase in the content of this polymer promoted a decrease in the fiber diameter. In vitro studies using PC12 cells revealed that the PCL/CS/PPy scaffold supports cell attachment and spreading, and it had an up to 356% increase in proliferation compared to pure PCL and PC12 neurite extension [180].

The combination of chitosan and alginate provides a hydrogel with improved mechanical properties and cell interactions. Alginate/chitosan (Alg/CS) hydrogel was used in the work of Salehi et al., 2019 [181] for the transplantation of olfactory ectomesenchymal stem cells (OE-MSCs) to promote peripheral nerve regeneration. Initially, a sciatic nerve injury was created in a rat model, and the Alg/CS hydrogel with OE-MSCs was positioned in the injured area and characterized. The hydrogel showed a porosity of 91.3 ± 1.27%, good blood compatibility, a swelling ratio of 379% after 240 min, and weight loss percentages of 80 ± 5.56% after 14 days. The results also showed that Alg/CS hydrogel with OE-MSCs could provide a suitable substrate for cell survival and increase regeneration compared to the control group and hydrogel without cells.

### 3.4. Chitosan in Wound Healing

Wound healing may be defined as a dynamic process that involves various molecules and cells, such as mediators, natural extracellular matrix (ECM), blood, and parenchymal cells [182]. Among the most frequently used natural polymers for the development of hydrogels applied in skin regeneration, chitosan (CS) stands out because it has characteristics such as antimicrobial action and reduced wound healing time [183]. Throughout the various stages of wound healing, a positive role is played by chitosan-based hydrogels. CS advances surface-induced thrombosis and blood clotting, and quickens coagulation in vivo. Besides, it influences platelet activation, the most significant component in blood clotting, and releases cytokines to improve the healing process [184]. The four stages for wound healing can be described as follows:

(1) Immediately after the injury, coagulation and hemostasis occur in the wound, which can avoid exsanguination and arrange a matrix for the invasion of cells necessary in the subsequent stages of healing [185].
(2) Soon after, the inflammatory phase of wound healing begins [186], controlled by inflammatory reactions moderated by cytokines, chemokines, growth factors, and their actions on cell receptors. The activation of intracellular signaling cascades contributes to cell proliferation, migration, and differentiation. Additionally, distinct types of cells (such as granulocytes and macrophages) are recruited by chemoattractant factors to initiate repair at the wound site [187]. In this process, an appropriate inflammatory microenvironment conducive to healing is formed by regulating cell activity and factors released by chitosan (CS)-based hydrogels.
(3) In 2 to 10 days after the injury, proliferation begins, featuring proliferation and migration in distinct cell types. The proliferative phase involves neoangiogenesis, development of granulation tissue and ECM, and re-epithelialization [188]. CS provides a non-protein matrix for 3D tissue growth and the activation of macrophages for tumoricidal activity. CS-based hydrogels can promote fibroblast proliferation, angiogenesis, and regular collagen deposition; enhance the natural hyaluronic acid (HA) level at the wound site; accelerate wound healing; and act in scar avoidance [6,96,189].
(4) In this phase, remodeling occurs, in which, by many enzymes and stress actions, the content and disposition of collagen fibers in the scar tissue are adapted to adjust to the physiological work, and this ends in the development of normal epithelium and maturation of scar tissue. A vital component of the dermal tissue present in chitosan is N-acetyl glucosamine (NAG), which is essential for repairing scar tissue [190].

Martins, et al. [191] investigated chitosan utilization in experimentally induced skin wounds in horses, and found that the chitosan membrane accentuated granulation tissue formation, emphasizing a potential repairing effect. Chitosan (CS)-based hydrogels are biocompatible, and in terms of wound healing, can afford a humid environment, offer security against secondary infections, eliminate wound exudate, incite quickly healing, and form soft scars [192]. On top of that, these materials can relieve factors that work against healing, present unique advantages due to their anti-inflammatory and antibacterial properties, curb the inflammatory reaction, and contain the infection, thereby providing a suitable microenvironment for healing [33,130]. CS-based hydrogels can also act as an obstacle to prevent the invasion and proliferation of microorganisms and provide structures for cell growth. Furthermore, since CS is a hemostat, it aids with natural blood coagulation and acts as a barrier for nerve endings, reducing pain [184].

Due to the antimicrobial characteristics and the capacity to transport extrinsic antimicrobial agents, chitosan is used to avoid or treat wounds and burn infections. Additionally, to improve wound healing, chitosan can be utilized as a slow-release drug-delivery vehicle for growth factors [193]. Some researchers have also reported that chitosan induces analgesia [194,195]. Allan and co-workers [195] verified that chitosan provided a refreshing, pleasant, and soothing topical sensation to open wounds. Okamoto, et al. [196] and Shigemasa and Minami [197] noticed that when animal wounds were covered with chitosan, accelerated wound healing was observed, along with a reduction in the treatment frequency, protection for the wound surface, and a decrease in or complete absence of pain.

Morphine, a commonly recommended drug for cancer pain relief, is regularly administered orally. Episodic and temporary pain is short-lived (40 min), frequently unpredictable, and can rapidly worsen, and due to slow pain control, treatment with oral opioid administration is not ideal. Parenteral administration provides faster control of pain relief but is not always an available, convenient, or preferred option. The nasal administration of painkillers offers faster pain relief. Therefore, it is worthwhile to adopt other non-oral routes of administration, such as transmucosal, nasal, or pulmonary, which can supply quickly pain alleviation [198]. The most convenient alternative method for releasing analgesic drugs seems to be the nasal route of administration. Morphine, however, being hydrophilic, is poorly absorbed via the nasal route. This problem can be solved by combining morphine with chitosan, a bioadhesive material that delays mucociliary clearance of morphine, permitting an extensive absorption time [199,200]. This morphine–chitosan release system can be of particular benefit to home-care patients [198].

Chitosan (CS) can act as a vehicle and enhance the action of some drugs used against pain. It has also been reported to enhance the bioavailability and dissolution properties of drugs with low solubility [201]. Maestrelli, et al. [202] evidenced CS’s efficiency in enhancing naproxen dissolution, a weakly water-soluble non-steroidal anti-inflammatory drug. Zerrouk, et al. [203] found a significant increase in naproxen analgesic activity after oral administration in rats using chitosan matrices. This formulation allowed a decrease in the dose required to obtain the analgesic effect, reducing the incidence of adverse effects.

Fráguas, et al. [204] performed CS chemical characterization to study this material’s healing effect on skin lesions of rats. They observed that in the evolution of the skin lesion healing, both in the control group and in the chitosan-treated group, superficial crust formation occurred until week 1 (7th day), from which there was a thickening of this crust. However, after week 2 (14th day), the crust detached from the lesion, allowing the drug to act directly on the wound and progressing to granulation and epithelialization tissue by week 3 (21st day). In this study, each lesion’s width and length were evaluated instead of its area, due to the slight variations in lesion shapes that occurred during the surgical procedure. Macroscopically, chitosan promoted an 81.4% reduction in the widths of the lesions, an elevated value compared to that observed in the control group (71.2%), thereby promoting a small increase in skin healing lesion.

However, chitosan has some limitations concerning mechanical strength and malleability for wound dressings, requiring blending with other polymers. This procedure is used to modify or obtain interesting properties, thereby developing a material that resists controlled degradation in a physiological environment [205]. For this purpose, it is mixed with other synthetic polymer hydrogels. In this sense, CS blends with synthetic polymers such as PVA [206,207], PVP [208], and PEO [209] have been developed and analyzed. Studies aiming to upgrade chitosan and PVA-based hydrogels’ characteristics and properties as dermal matrices are increasingly needed. They have been highlighted by the numerous possibilities of material combinations to treat injured tissues [210].

Fan, et al. [211] studied gamma radiation formulation and characterization of CS/gelatin/PVA-based hydrogels as dressings in injured tissues. They found through blood clotting results that the hydrogels analyzed rapidly absorbed blood from the damaged tissue and adhered to the dermal surface, blocking broken blood vessels and stimulating platelet release, which can promote blood clotting. The hydrogels elaborated on in this research have promising clinical potential to be applied to skin wound coating.

Chitosan can also interact with negatively charged polymers, forming polyelectrolyte complexes (PECs). The anionic polysaccharides alginate or xanthan are utilized for this purpose, creating materials with superior properties to those presented by these polymers alone. In this sense, Souza, et al. [212] incorporated erythromycin into hydrogels with chitosan-alginate and chitosan-xanthan polyelectrolyte complexes for application in the treatment of skin lesions. Owing to the relatively large size of the erythromycin molecule, its release from the matrices was slow, requiring less frequent changes of the wound dressings and resulting in less traumatic and more comfortable treatment for the patient.

The response of the host tissue to CS-based implants is an essential biological property. It was found that, in general, once these materials were implanted, they aroused a minimum foreign body reaction, with few or no fibrous encapsulations. The generation of granulated tissue stimulated by incorporating the material implanted in the host is usually affiliated with quick angiogenesis and is related to the healing response [6].

Suh and Matthew [90] reported that the stimulation of local cell proliferation and the incorporation of the material implanted with the host tissue can be performed by chitosan and its fragments in immune cells, since chitosan has favorable properties to promote accelerated dermal regeneration and quick wound healing, presenting itself as a suitable material for applications ranging from mere wound coverings to elaborate artificial skin matrices.

It can be concluded that chitosan’s aforementioned characteristics justify this material’s application in tissue regeneration systems. Concerning wound healing applications, CS-based hydrogels can supply a humid wound environment, protect against secondary infections, accelerate healing, and lead to soft scars. In this context, Table 1 lists several recent studies over the last 10 years on chitosan uses in tissue engineering and wound healing, showing the relevant results of each study.

## 4. The Use of Chitosan as a Drug Delivery System

Several devices have been proposed to maintain therapeutic levels, maintain long-term drug concentration in the body for disease treatment, promote greater adherence, increase administration intervals, and decrease side effects, among other advantages [217]. Such a strategy involves controlled drug release systems commonly referred to as drug delivery systems (DDSs) and requires broad interdisciplinary, involving, mainly, knowledge in pharmaceutical sciences, polymer science, colloid chemistry, physicochemical, and molecular biology [218].

Drug delivery systems (DDSs) can be described as methods through which a wide variety of drugs can be delivered to desired absorption sites, such as organs, cells, and tissues, while aiming to enhance the drugs’ pharmacological activities, and surpass difficulties such as poor solubility, low bioavailability, drug aggregation, limited distribution in the body, and absence of selectivity [219]. The search for new controlled drug release systems has been very relevant to establishing more efficient therapeutic alternatives that can administer drugs more safely and minimize side effects [220].

**Figure 4:**
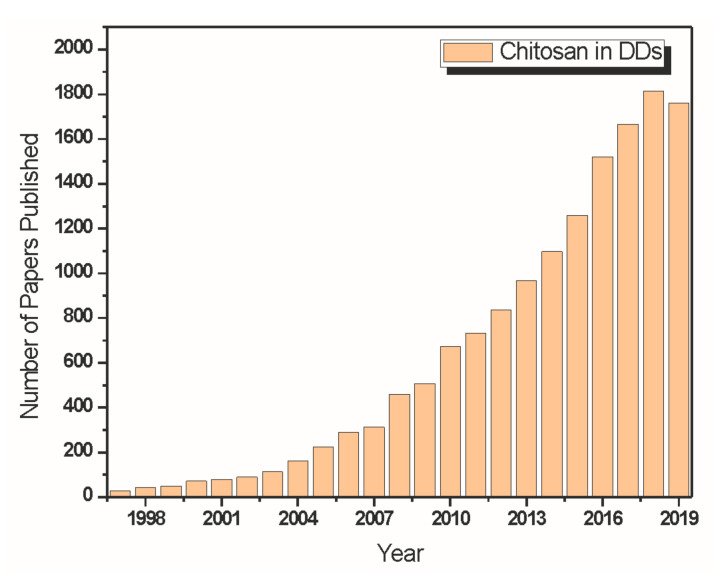
Literature data extracted from the Web of Science Core Collection about papers that address chitosan (CS) uses in drug delivery systems (DDSs).

Chitosan (CS) is one of the most functional natural biopolymers widely used in the pharmaceutical field due to its biocompatibility and biodegradability. These characteristics lead to its promising potential for use as a carrier for drug delivery, especially in the last two decades [221]. Figure 4 is a graph obtained by a statistic extracted from the Web of Science Core Collection about the number of papers published addressing chitosan in drug delivery systems over the last years.

Chitosan (CS) granules can swell and float in an acidic medium (pH 1.2), allowing extensive use of this material in sustained-release preparations of many drugs. From a photographic analysis of the stomach of a rabbit three hours after oral administration of CS granules, the swelling of these granules was observed, which were held in the stomach for at least this duration. Additionally, CS granules have a gel-forming characteristic in a low pH range, which, added to chitosan’s antacid and anti-ulcer activities, can prevent stomach irritation from medications [222,223].

However, some authors reported an agile rate of stomach dissolution of chitosan as one disadvantage of using this polymer in controlled release formulations for oral administration. Once at small pH values (beneath the pKa value), this natural polysaccharide is positively charged; this cationic character can be used for interaction with anionic polymers to afford a more precise controlled drug delivery, forming polyelectrolyte complexes obtained via electrostatic interactions of the anionic groups present in aqueous solutions with the cationic amino groups present on the glucopyranose units of chitosan at the C-2 position. For the formation of those complexes, the primary binding force is related to the coulombic force, and they have been frequently studied and investigated for pharmaceutical applications due to exhibiting favorable physicochemical properties and maintaining the biocompatible properties of chitosan [224,225,226,227,228].

Takahashi, et al. [229] studied the chitosan–sodium alginate (CS–AL) and chitosan–sodium acrylate (CS–AC) complexes. The authors concluded that the flexibility or rigidity of polymer chains was the main feature responsible for the variations noticed among these systems. In terms of changing pH values, the CS–AL complex is steady to these changes, and the CS–AC complex is pretty sensible, which should be considered when applying more accurately controlled drug delivery systems.

Compared to other polymer-coated liposomes, CS-coated liposomes also presented better in vitro adhesion properties for rat intestine when used as an oral DDS. Additionally, in vivo studies of chitosan-coated liposomes containing insulin orally administrated in rats showed a significant reduction in this animal’s blood glucose levels 12 h after administration [230].

CS is a biopolymer that has good miscibility with clay and can easily intercalate in interlamellar spaces. The intercalation results from the polycationic and hydrophilic nature of this polymer in an acidic medium, as found elsewhere [231,232,233]. This hybrid material favors the permeability and bioavailability of drugs by the body [232,234].

Tavares, et al. [235] prepared chitosan–montmorillonite films, in the mass proportion of 20 wt.% clay without and with the presence of ibuprofen, an important hydrophobic non-steroidal anti-inflammatory drug whose controlled release can improve its therapeutic efficacy and reduce the side effects caused to the central nervous system and the gastrointestinal tract by its frequent or high-dose use [236]. These bio-nanocomposites were homogeneous in terms of drug dispersion. Additionally, they showed a slower and more controlled release rate of the drug, indicating that the development of this bio-nanocomposite is promising for DDSs.

The design of effective and secure chitosan-based dosage forms has received considerable attention [237]. One of them is the solid-lipid nanoparticles (SLN), which have made drug delivery systems with fewer side-effects, better targeting, and protection from enzymes [238]. The improvement of drug absorption via the paracellular route through the neutralization of fixed anionic sites in the tight junctions among mucosal cells promoted by CS has also been reported [44,239].

The nasal cavity is considered an advantageous route for drug delivery due to the absorption promoted by the high surface area with a highly vascularized subepithelial layer and the prevention of liver first-pass metabolism. Chitosan was evaluated as a nasal delivery system and was found to facilitate the passage across the nasal mucosa of large hydrophilic molecules, such as salmon calcitonin and insulin [199]. The presence of CS can increase the delivery system’s contact time with the nasal mucosa and arrange the potential to increase the bioavailability of drugs embodied in these systems [240].

In this context, improved absorption by chitosan of peptides and proteins across nasal [199] and also intestinal epithelia [44,241] has been demonstrated, which is based on the aforementioned charged interaction, obtained by an ionic interaction among the mucoadhesive properties of this polymer and the negatively charged cell membrane [239].

From an analysis of the literature, Peppas and Buri [242] concluded that several characteristics of the polymer are required for mucoadhesion, such as sufficient chain flexibility, the existence of solid hydrogen-bonding groups (−OH, −COOH), elevated molecular weight, strong anionic charges, and properties of surface energy that favor the spread onto mucus. In this context, chitosan appears as an interesting candidate, and its mucoadhesive properties were studied, aiming for a possible oral application since it was found that positively charged polymer hydrogels would be able to promote by electrostatic interactions other forces of molecular attraction with negative charges mucosal surfaces [241].

Chitosan’s solubility in acidic solutions may explain the short and weak mucoadhesion of this polymer in an artificial gastric fluid, a factor that may be overcome by blending it with other non-soluble polymers or drugs or by chemical cross-linking [241]. However, according to Park, et al. [243], mainly in a neutral or slightly alkaline medium, cationic polymers are probably superior mucoadhesives, a desirable adhesion factor in the small or large intestine. Chitosan has minimum swelling in the artificial intestinal fluid, which can be explained by the low solubility in water. The replacement of free amino groups with short alkyl chains enhances the pK (kinetic constant) and promotes these groups’ ionization at an elevated pH value. In turn, the swelling will improve and probably also mucoadhesion.

The mucoadhesive properties of various natural or partially modified polymers were investigated by routinely measuring in a saline medium the detachment force of swollen polymeric films from the intestinal mucosa of pigs. Hydroxypropylcellulose and carboxymethylcellulose displayed nearly none mucoadhesion. At the same time, chitosan proved to be quite mucoadhesive compared to polycarbophil (a poly(acrylic acid) copolymer freely cross-linked with divinyl glycol that presents a high molecular weight and excellent mucoadhesive properties) [241].

Utilizing an in vitro model based on Caco-2 cells monolayers derived from a human colon carcinoma cell line, the ability of the mucoadhesive polymers carbomer and chitosan-glutamate to increase intestinal peptide drug delivery was investigated. The polymer preparations were applied to the apical side of the Caco-2 cell monolayers. The results showed a significant decrease in the transepithelial electrical resistance (TEER) promoted by the polymers, allowing them to transport hydrophilic compounds through these cell monolayers [239].

The insolubility of chitosan in neutral physiological fluids can be overcome by modifying the active groups on its backbone, such as −OH, −NH_2_. This ameliorates the DDS properties when compared to unmodified chitosan. Chitosan derivatives preserve the major polymer demeanor and have thermoresponsiveness and pH dependence, allowing them to adapt the polymer’s behavior to the requirements of the application. CS and their derivatives can synergistically create nanoparticles with different drug carriers. Two polymers with different charges give rise to a polyelectrolyte complex that forms a matrix in which drug molecules can be entrapped. However, the coating of one carrier with the other can also occur [244,245].

N-carboxymethyl and N-trimethyl chitosan were also studied for DDSs. Their characteristic of adhering to mucosal surfaces is advantageous for the mucosal release of drugs [61,130,246,247,248]. The mucoadhesive properties of N-trimethyl chitosan (TMC) have been attributed to an interaction between its cationic groups and the anionic residues of sialic and sulfonic acid from mucin. Since it can open the tight junctions between epithelial cells, TMC promotes the transport of hydrophilic and peptide molecules through the paracellular route, and its amphiphilic nature allows it to be assembled into vesicles that can be used for the preparation of various systems for the release of drugs and genes through different routes, such as oral, ocular, nasal, and rectal [245].

Kotzé and co-workers [249] synthesized a chitosan (CS) derivative partly quaternized N-trimethyl chitosan chloride (TMC), and utilizing Caco-2 cell monolayers, studied the action of this material on the intestinal epithelial cells permeability, and the values obtained were compared with those of CS hydrochloride and CS glutamate. The results evidenced that the CS salts and the TMC increased the carry of extensive hydrophilic compounds by opening tight junctions to allow paracellular transport, being considered a possible absorption improver for the intestinal absorption of buseriline and nasal absorption of insulin and calcitonin, and its structural characteristics, charge and charge density, are important factors that determine the use of these materials for this application.

Among chitosan derivatives, quaternary ammonium salts present a permanent positive charge, making them widely used to deliver drugs and genes through different administration routes. They have considerable biocompatibility, low toxicity, biodegradability, improved mucoadhesiveness, and solubility in water regardless of pH, promoting increases in the release and permeation of drugs across biological barriers and neutral/alkaline environments [245].

N-2-hydroxypropyl trimethyl ammonium chloride chitosan (N-2-HACC) and N,O-carboxymethyl chitosan (CMC) were synthesized as an adjuvant and a delivery carrier for the vaccine antigens of Newcastle disease virus (NDV) and infectious bronchitis virus (IBV). Chickens were immunized via intranasal administration to evaluate the immune response, and the results showed these NPs exhibited lower cytotoxicity, higher stability, and induced humoral, cellular, and mucosal immune responses. As a result, the chickens were protected against the infection of highly virulent NDV and IBV, indicating that the N-2-HACC-CMC NP is an efficient adjuvant and delivery system for mucosal vaccine immunity [250].

The hydrolysis of chitosan gives rise to a low molecular weight product that is extremely soluble in water (more than 50% (*w*/*v*), called low molecular weight chitosan (LMC). LMC received much attention from the pharmaceutical industry, as it can increase the absorption rate of a drug that presents low water solubility by enhancing the dissolution rate of the drug, making the drug’s surface hydrophilic by dispersing it into chitosan [201].

Low molecular (LM) chitosan and alginate were investigated for DDSs [201] for betamethasone, phenytoin, prednisolone, diazepam, and digoxin. Results showed that LM chitosan and LM alginate enhanced the dissolution rates of drugs owing to the increase in the wettability and the curtailment of crystallinity and crystal size.

Chitosan is available as low molecular weight chitosan (CSL), medium molecular weight chitosan (CSM), and high molecular weight chitosan (CSH) depending on the degree of de-polymerization, with different cationic charge densities and viscosities. Atorvastatin (ATR) is a drug that undergoes high intestinal clearance and first-pass metabolism, resulting in low absolute and systemic bioavailability. In this context, a biodegradable, sustained release formulation of ATR can be obtained by finding an ideal concentration/molecular weight/cationic charge of chitosan for the size reduction and stabilization of nanocrystals [251,252].

In this sense, Kurakula et al. [252] developed a novel oral chitosan-based atorvastatin (ATR) nanocrystal formulation using a combination of anti-solvent and probe sonication methods to improve the intrinsic solubility, dissolution release, and bioavailability of ATR. The method used proved efficient through high drug loading, better size reduction, and cost-effectiveness. The influences of cationic charge densities of chitosan (low CSL, medium CSM, high CSH molecular weights) were investigated, and low-cationically-charged chitosan proved to be effective in size reduction, enhancement of solubility, and stability of nanocrystal formulations, thereby being considered a promising strategy to formulate oral sustained release dosage forms to improve the bioavailability of major Biopharmaceutical Classification System II/IV drugs.

A solid complex was also prepared from the mixture of appropriate amounts of indomethacin (IM), a non-steroidal anti-inflammatory drug employed in arthritis treatment, and LM chitosan (C-I), and the results evidenced that the solubility of the IM enlarges with enhancing quantities of LM chitosan. As chitosan is a basic polysaccharide, chitosan’s buffering capacity may have influenced the increasing solubility of the acidic drug IM [253].

Afterward, for oral administration of granules made of a 1:2 mixture of drug and chitosan (CS) in rabbits, prolonged absorption of indomethacin (IM) was reported [222] compared to the oral administration of a conventional commercial capsule, which implied an utmost level of the plasma concentration after one hour. When administering granules with a 1:2 mixture of CS and drug, there was not an accentuated peak of plasma concentration, but a sustained level of IM in the plateau. This resulted from a slow rate of granule release, and consequently, the extended residence time in the stomach. Thus, the chitosan granules proved to be an interesting choice compared to conventional commercial capsules to reduce the peak plasma concentration and conserve the plasma IM concentration.

The use of encapsulated indomethacin (IM), through polyacrylamide-grafted chitosan microspheres cross-linked with glutaraldehyde, can control the liberation of indomethacin depending on the crosslinking density. Additionally, the initial release of indomethacin is attributed to the polymeric chain relaxation process, occurring later than the completely swollen polymer and being mainly administered by the molecular diffusion phenomenon [254].

The microflora, which is colon-available, can degrade chitosan. Due to this property, chitosan can be considered a polymeric matrix specific for colon drug release [255,256,257]. Specific oral release of sodium diclofenac into the colon by chitosan succinate and chitosan phthalate matrices has also been favorably exploited [255].

The provision and maintenance of an adequate drug concentration at the local of action is the main problem of ocular therapy. This is because less than 5% of the drug applied through a topical ophthalmic formulation enters the cornea and reaches the intraocular tissues, thereby implying the need for frequent instillations to achieve the therapeutic effect—a thing that would be significantly improved by increasing the drug’s retention in the precorneal area and the penetration of the drug through the cornea. In this context, the application of chitosan in ophthalmology has been extensively researched, as it is a material that is considered an excellent drug carrier. It can easily form stable and appropriately sized nanoparticles, with quick and mild procedures that do not induce any irritation. Thus, a mucoadhesive biopolymer would be able to secure the encapsulated molecule, improve its mode of action, and attenuate any existing danger [244].

Besides, the polymer’s primary amine group’s protonation, when dissolved in an acidic environment, leads to an ionic interaction among the negatively charged ocular mucus and chitosan. This mucoadhesiveness results in enhancing the nanoparticles’ residence time on the mucin, prolonging the drug contact time with the cornea. Therefore, since there is a significant increase in the drug bioavailability in the eye, its penetration into the inner layers is facilitated. Owing to the positive surface charge and the capacity to increase the passive drug diffusion, chitosan nanoparticles can penetrate both the conjunctiva and the cornea [244].

Chitosan and poly-L-lysine(PLL)-coated poly-ε-caprolactone (PECL) nanocapsules were studied as ocular carriers, and the results showed that, compared to commercial eye drops, there were considerable increases in the IM concentrations in the corneas and aqueous humor of rabbit eyes. Additionally, chitosan (CS)-coated nanocapsules presented good eye tolerance and are considered an advantageous method to improve drugs’ ocular bioavailability [258].

The main monomeric unit of chitosan is β-(1→4)-*N*-acetyl-D-glucosamine, and glucosamine sulfate, a pharmaceutical derivative of the naturally found amino monosaccharide glucosamine, has been used to treat rheumatic diseases including osteoarthritis [18]. Additionally, the postponement in the long-term progression of knee osteoarthritis regarding modifications and symptoms of the joint structure by glucosamine sulfate has been proven.

Table 2 shows some chitosan-based systems’ applications, as DDSs mentioned by studies in the last 10 years, the type of drug used for loading, and the relevant results presented by these studies. Once chitosan has chemical groups that can undergo modifications, this polymer represents an option for a controlled drug release agent. For this purpose, this biopolymer can be combined with other polymers or even with clay, presenting good miscibility, and this combination favors the permeability and bioavailability of drugs by the body.

### 4.1. Chitosan in Cancer Treatment

The study of the medical or therapeutic potentials of several natural products, including cancer prevention and treatment, has been carried out since the induction or maintenance of immune responses; in addition, the direct targeting of tumor cells through epigenetic modifications or apoptosis mechanisms can be performed by some of them [273,274].

**Figure 5:**
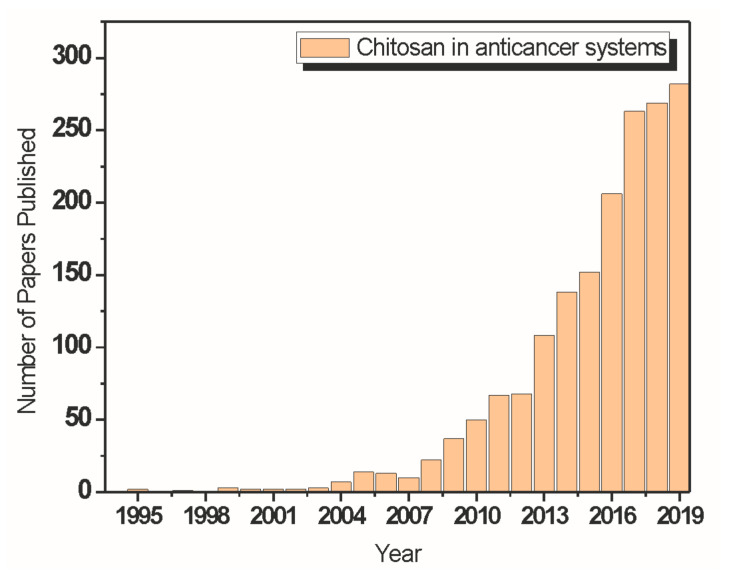
Literature data extracted from the Web of Science Core Collection about papers that address chitosan (CS) in anticancer systems.

Due to chitosan (CS) properties such as bioavailability, mucoadhesivity, biocompatibility, biodegradability, non-toxicity, and low immunogenicity, this biopolymer has drawn attention in pharmaceutical and biomedical applications. Additionally, CS is soluble in dilute acidic solutions since it has primary amino groups (pKa = 6.3) [9], and it is degraded in vertebrates by lysozyme and bacterial enzymes in the colon, making CS an exceptional carrier for anticancer drugs. In this context, Figure 5 is a graph obtained by a statistic extracted from data from the Web of Science Core Collection about the number of papers published addressing chitosan in anticancer systems over the last years.

To eliminate the anticancer drug’s adverse effects, various drug delivery systems (DDS) were generated with the aim of targeting tumor cells and releasing active biomolecules at a particular infection location [275,276]. Chitosan is a positively charged polymer at acidic pH due to protonable amine groups, which allows electrostatic interactions with polyanions when this polymer is utilized as a carrier to deliver genes or drugs [277]. Rao, et al. [278] showed that chitosan could eliminate tumor-initiating cancer stem cells by doxorubicin (DOX) delivery to the tumor microenvironment. A six-fold increase in the DOX cytotoxicity was observed compared to the use of free DOX for eliminating CD44^+^ cancer stem-like cells residing in 3D mammary tumor spheroids. Besides, a reduction in the tumor’s size in an orthotopic xenograft tumor model with no evident systemic toxicity was found. The direct antitumor activity of chitosan was evidenced in the work of Qin, et al. [279], which is capable of curbing the increase of sarcoma 180 tumor cells in mice.

While aiming to improve the targeting and cytotoxic effects of ellagic acid (EA) on colon cancer cells, EA was encapsulated in chitosan (CS) and then coated by eudragit S100 (ES100) microparticles in the work of Alhakamy et al. [280]. Results indicated that the generated material exhibits improved colon targeting and enhanced cytotoxic and proapoptotic activity against HCT 116 colon cancer compared with raw EA administration.

A newly discovered phenomenon in which innate immune responses may be induced by the natural product chitosan (CS), facilitating the cross-talk among DCs and NK cells, was described by Li and co-workers [11]. They found that DCs are directly activated by chitosan, improving the effector functions of human NK cells, promoting these cells’ survival, and enhancing their cytotoxicity-averse to leukemia cells. The use of a B16 melanoma mouse model showed better in vivo antitumor activity, showing that CS may be clinically utilized to avoid or treat infectious diseases, and even cancer.

Microspheres produced from a polymeric blend of chitosan and PVA were promising for the controlled release of cisplatin Souza, et al. [281]. The dimensional and morphological characterization showed spheres with low porosity and irregular surfaces that were stable during the adsorption process.

Hybrid lipid polymer nanoparticles can be prepared to try to solve the main disadvantages of chemotherapeutics, such as non-specific and uncontrolled delivery of drugs, since lipids have better biocompatible properties and polymers have structural benefits—factors that can be achieved when utilizing this system for controlled drug delivery at tumor sites [282,283]. In this context, safer drug delivery to tumors can be achieved by encapsulating cisplatin in a lipid polymer system [284].

Hybrid lipid–chitosan nanoparticles loaded with cisplatin have been formulated with several proportions of chitosan to lipid in a new study [283] by the single-step ionic gelation method based on the ionic interaction between these oppositely charged polymers to achieve the ideal particle size, encapsulation efficacy, and controlled release pattern. The drug release regulation is given by the chitosan matrix and the lipid layer that avoids the pouring of drugs, and the default of an explosive release of cisplatin was attributed to its imprisonment in the inner polymer layer and outer lipid covering. Cell viability studies confirmed the cytotoxic effect on the A2780 ovarian cancer cell line over a 48 h period. The cellular uptake studies showed an increase in the uptake of LPHNPs. Additionally, cisplatin’s controlled release behavior with a longer average residence time and a longer half-life was evidenced through in vivo pharmacokinetics studies in rabbits. Toxicity studies in rats supplied a safety profile of LPHNPs. Afterward, the instant immersion in the medium in vitro dissolution study demonstrated that neither of the formulations presented an explosive cisplatin release. However, a sustained release took place over 24 h from the lipid-polymer complex. The results of this study pointed out that LPHNP loaded with cisplatin is a propitious platform for the controlled release of this drug in cancer therapy.

The release of taxol by a N-lauryl carboxymethyl chitosan (LCC) matrix, containing hydrophilic and hydrophobic groups, was studied to treat cancerous tissues. Taxol solubilized in LCC formed micelles of particle size smaller than a hundred nanometers, capable of passively targeting tumors [285].

There are four main advantages of using nanoscale drug delivery systems (DDSs) for chemotherapy, namely:

(1) Supporting in the solubilization of hydrophobic drugs in aqueous media by polymeric nanocarriers [286];
(2) Longer blood circulation times for nanocomposites with the appropriate size (70–200 nm), which increases permeability and the retention effect (EPR) to passively target tumor tissue;
(3) Microenvironment-responsive properties of DDSs can be obtained by grafting a nanocarrier with stimuli-responsive materials, used to guarantee the drug release solely at the target site;
(4) The target side effects that can be minimized by the functionalization of nanomaterials with targeting ligands to obtain particular binding and uptake by tumor cells [287,288].

The carriers for loading anticancer drugs are commonly composed of amphiphilic polymers or hydrophilic biopolymers. In this scenario, due to low toxicity, distinguished stability, and easy accessibility and modification presented by chitosan nanoparticles (CSNPs), they have attracted considerable attention as anticancer drug delivery carriers [275,289]. However, the toxicity of nanosized particles of CS and its derivatives should not be ignored when utilized in the delivery of drugs and genes since nanoparticles can enter systemic circulation from the nasal cavity, gastrointestinal tract, or alveolar sacs, causing various levels of toxicity for the human body. Moreover, to achieve a specific targeting therapy, structural modification of chitosan is carried out. This may lead to some toxicity depending on the toxicity of the reactant(s) used [290,291].

The characteristics of the parental material used to prepare the nanoparticles, cell interaction, and particle size are associated with the level of toxicity of the CSNPs [292]. Huang, et al. [293] related a decrease in the cell uptake capacity and binding affinity of CSNPs with a reduction of the DD of the parent polymer and molecular weight (Mw). When the Mw decreased from 213,000 to 10,000, there was a 26% drop in cell uptake. Additionally, there was a 41% reduction in this property when DD decreased from 88% to 46%.

Since the liver is the detoxifying organ, the hepatic toxicity of CSNPs is of concern because they show unwanted effects on hepatic cells. Loh, Yeoh, Saunders and Lim [292] also evaluated the hepatotoxicity of CSNPs, and the cell toxicity analysis results revealed that human liver cells could tolerate 0.5% *w*/*v* of CSNPs (18 ± 1 nm, 7.5 ± 1.0 mV in culture medium) for up to 4 h. The integrity of the cell membrane was compromised when chitosan nanoparticles reached concentrations above 0.5% *w*/*v*, as demonstrated by the leak into the alanine transaminase’s extracellular medium.

#### 4.1.1. Therapeutic Metal Ions (TMIs)

Combining chitosan with metal and metal oxides NPs is a strategy to increase these polymer characteristics, which is possible due to their free amino groups’ connections with these NPs, forming strong complexes [294]. In this context, ZnO, TiO_2_, and mainly Ag NPs, stand out among the many metals (oxides) used in various studies combined with chitosan.

One of the main advantages of using ZnO nanoparticles to treat cancer is the preferential cytotoxicity inherent against cancer cells in vitro. Additionally, a high degree of selectivity toward cancer cells by zinc oxide NPs has been demonstrated by many studies, including the ability to overcome the therapeutic indices of some chemotherapeutic agents usually utilized in resembling ex vivo studies [295,296].

Carboxymethyl chitosan (O-CMCS) is an amphiprotic ether derived from chitosan that has received considerable attention in cancer therapy. Water-soluble nanocomposites (NCs) based on O-CMCS with nanostructured zinc oxide (n-ZnO) were synthesized by the ex-situ grafting method for the delivery of curcumin (Cr) aiming at anticancer effect by Upadhyaya et al. [297]. The drug trapping efficiency was 74%, and the in vitro drug release study carried out at 37 °C at pH 4.5 and 7.4 showed initially slow release, followed by sustained release. The MTT assay showed preferential and higher toxicity of Cr/O-CMCS/n-ZnO NCs against cancer cells (MA104) compared to normal cells (L929). Cell uptake by FACS revealed an uptake dependent on the concentration of NCs. The authors concluded that the Cr/O-CMCS/n-ZnO nanocomposite consists of a promising soluble nanomatrix that offers an efficient anticancer therapy strategy.

ZnO nanoparticles reduced by Amorphophallus paeoniifolius (Ap) coated with chitosan (CS) were produced in the work of Anitha et al. [298] for controlled and localized drug release to investigate the anticancer potential against the MCF-7 cell line (breast cancer cells). The MTT assay showed that, in a dose-dependent manner, spherical and cubic nanocrystals were lethal against MCF-7 cells, which shrunk and exhibited nuclear damage. Additionally, the microscopic bright field study showed the apoptotic morphology of treated and control MCF-7 cells. Fluorescence staining (A/O: EB and DAPI) also revealed apoptotic characteristics, such as chromosomal condensation, different intracellular location of the intermediate filaments, nuclear fragmentation, and confirmed the dose-dependent CS-Ap-ZnONPS-induced apoptosis. This study revealed that the ZnO nanoparticles reduced by *Amorphophallus paeoniifolius* coated with CS had anti-cancer properties with less toxicity for normal cells and more significant toxicity for MCF-7 cells in vitro. Thus, CS-Ap-ZnONPs proved to be an excellent anticancer agent in controlling the proliferation of the breast cancer cell line in vitro by inducing apoptosis.

Environmentally friendly intelligent vehicles for colon-specific drug delivery using biopolymers sensitive to the pH, carboxymethylcellulose (CMC), and chitosan (CS) were developed in the work of Sun, et al. [299]. ZnO nanoparticles were embodied in the CMC beads and then covered with a chitosan layer through a self-assembly technique to create core-shell polyelectrolytes complexes to surpass the debilities of CMC carriers, such as an explosive drug release and low mechanical properties. The bio-nanocomposite beads of ZnO/CMC/CS with reduced porosity were effectively loaded with an anticancer drug, 5-fluorouracil (5-FU). They showed pH-sensitivity, biodegradation capacity, and self-sustaining release behavior, relying on the content of CMC, CS, and ZnO nanoparticles.

The physical form and appearance of metallic or inorganic compounds have powerful repercussions for the therapeutic efficiency of ZnO, even though it is considered fatal for cancer cells [300]. Some works evidenced that zinc oxide’s toxicity significantly influences cancer cells, depending on time and concentration, with zinc oxide in the nanoparticulate form presenting advantages when compared with the free metal form [301]. The metal ion delivery in the intracellular environment is primarily responsible for the cytotoxic effect of Zn^2+^, which is only feasible by the efficacious internalization of the metal ions in the cell compartment. The released Zn^2+^ damages bacteria by amino acid metabolism, active transport inhibition, and disrupting the enzyme system [302,303,304,305]. Therefore, for the death of cancer cells, nanoparticulate zinc (ZNP) may be advantageous, and the use of a tactic that can enhance the intracellular uptake of zinc ions may present more benefits [306]. However, the limited cellular internalization of the metal ions by ZNP in cancer cells, which results from its opposing surface charge, can be improved by modifying its surface with naturally derived chitosan to enhance cellular internalization [307,308].

The anticancer efficacy of chitosan-assembled zinc oxide nanoparticles (CZNPs) against cervical cancer cells was investigated by Wu and Zhang [309]. The significant cytotoxicity of ZNP and CZNPs in cervical cancer cells was mostly assigned to the generation of reactive oxygen species (ROS) in cancer cells and was found to be dependent on the concentration. The major cause of the cell-killing effect of ZNP was attributed to apoptosis through the apoptosis assay and reaffirmed by the nuclear chromatin assay. In addition, enhanced red fluorescent cells for chitosan-assembled zinc oxide nanoparticle to treat cancer cells were demonstrated in the live/dead assay. In general, the authors concluded that the transmission of cytotoxicity to cervical cancer cells through the metal oxide existing in the nanoparticulate dimensions would be convenient.

Chakra, et al. [310] studied the antibacterial and anticancer activities of a nanocomposite synthesized mixture of ZnO and TiO_2_ NPs. It was observed that the synergistic activity of the nanocomposite against bacteria and cancer was maximal when compared to the isolated compounds.

Nanocomposites fabricated in combination with chitosan and titanium dioxide (TiO_2_) has significant effects on photocatalytic activity, mechanical strength, chemical resistance under UV, biocompatibility, solubility in acidic/alkaline media, and antimicrobial behavior. Moreover, the enhancement of the biological, physical, and mechanical properties of chitosan by inorganic compounds containing TiO_2_ has also been reported [311,312,313,314]. CS-TiO_2_ composites have low cytotoxic effects on distinct cell culture lines and improved antibacterial and antiviral properties due to the photocatalytic activity of TiO_2_ [315].

An amperometric immunobiosensor for detecting α-fetoprotein utilizing an Au/CS/TiO_2_-graphene (Gr) composite was developed by Huang, et al. [316]. α-fetoprotein consists of an oncofetal glycoprotein, one of the most widely used clinical cancer biomarkers. Electrostatic adsorption of negatively charged AuNPs can occur on the positively charged chitosan/TiO_2_-Gr composite film, and then, for the α-fetoprotein (AFP) assay, immobilization of the α-fetoprotein antibody occurs. According to the results, this immunobiosensor was considered a new tactic for AFP detection with great sensitivity, bioactivity, and selectivity with suitable storage stability. Thus, the authors considered that the development of biosensors from the Au/CS/TiO_2_-graphene composite aimed at the detection of various antigens or compounds is an interesting alternative.

The characteristics presented by chitosan, such as biocompatibility, safety, biodegradability, bioadhesivity, non-toxicity, and promotion of drug absorption, make this polymer a good option for the transport of silver nanoparticles (AgNP) [317]. Although the effectiveness of AgNP as an alternative antimicrobial material has been proven, exhibiting high toxicity to a broad range of micro-organisms and cell lines [318,319,320], it is necessary to use a vehicle for the sustainable transport of nanoparticles to specific locations; it is worth highlighting the excellent results obtained with the use of chitosan (CS) hydrogels in drug transport [321,322]. Xie and co-workers [214] reported that Ag nanoparticles integrated into the chitosan hydrogel networks can enhance the CS hydrogel’s antibacterial properties and reinforce its mechanical properties.

Nanocomposites can be used to promote a more significant improvement in CS’s antibacterial properties and help in the cancer treatment. Since silver (Ag) nanoparticles present excellent antimicrobial properties, they are an obvious choice for increasing the antibacterial activity by adding a filler [323,324,325]. AgNPs are also toxic to cancerous cells [326] and are also feasible aspirants in drug delivery [327,328,329]. However, the tendency presented by this nanoparticle for agglomeration during the reduction of its metallic salts makes its use in the biological field inappropriate. Stabilizing agents/surfactant is an excellent approach to avoid particle agglomeration [330,331,332]. Nivethaa, et al. [333] proved that chitosan (CS) has the function of a stabilizing agent, by acting as a material that provides places for drug molecule encapsulation.

The CS nanocarrier (NC)-based delivery of Ag NP development to mammalian cells for apoptosis induction with an inferior content of NPs has been reported by Sanpui, Chattopadhyay and Ghosh [326]. Morphological analyzes and biochemical assays were used to examine the Ag–CS NC system’s cytotoxic efficiency in human colon cancer cells (HT 29). A 50% decrease in HT 29 cells’ viability was achieved for an Ag NP concentration of 0.33 μg mL^−1^, as demonstrated by the cell viability assay. The depolarization of the mitochondrial membrane potential showed the implication of the mitochondrial pathway of dying of cell in the Ag–CS NC-induced apoptosis. The up-regulation of caspase 3 expression considered in the generation of oligo-nucleosomal DNA “ladders” in Ag-CS NC-treated cells was demonstrated through real-time quantitative RT-PCR analysis, indicating the importance of caspases in the actual apoptotic process. Ag-CS NCs treatment increased intracellular ROS production, indicating that the increase in apoptosis induction in HT 29 cells can be obtained by oxidative stress and the classic caspase signaling pathway. The authors concluded that the approach of using a meanly low content of Ag NPs ingrained in CS nanocarriers is remarkable for cancer therapy when compared to the use of free Ag NPs.

Due to the intrinsic properties against cancer presented by multi-walled carbon nanotubes (MWCNTs), they interfere in microtubules’ dynamics, triggering antiproliferative, anti-migratory, and cytotoxic effects in vitro that inhibit tumor growth in vivo [334]. The potential use of MWCNTs as antitumor agents, using novel cytotoxic mechanisms, can be proven by maintaining their effects on tumors unaffected by traditional microtubule-binding chemotherapies, such as Taxol^®^ [335]. MWCNT are being developed as a new DDS for lung cancer therapy, exhibiting many exceptional features—ultralightweight, a diameter smaller than one hundred nanometers, a high surface area, length of 0.5 to 20 µm, an internal hollow space, greater capacity for cell penetration, neutral electrostatic potential, high capacity for drug encapsulation, and cancer cell targeting [336,337,338]. Improved cell penetration into lung cancer cells without causing damage to healthy cells was achieved through efficient cancer targeting by MWCNT, which also suits a multifaceted platform for delivering a diversity of diagnostic and therapeutic agents [339,340,341].

Nanocomposites of chitosan/silver (CS/Ag) and chitosan/silver/multi-walled carbon nanotubes (CS/Ag/MWCNT) encapsulated with 5-FU using the chemical method were sintered in the work of Nivethaa, Dhanavel, Rebekah, Narayanan and Stephen [333] aiming to study the cytotoxicity and release profiles of the systems towards MCF-7 cell line. Morphological studies indicated the formation of a polymeric matrix nanocomposite, and the encapsulation of 5-FU in these systems was indicated through XRD, SAED, and elemental mapping. A sustained and prolonged release was evidenced in the case of the CS/Ag/MWCNT nanocomposite. The cytotoxicities of nanocomposite systems were studied using MCF-7 cell lines, and a more significant cytotoxicity was verified for CS/Ag/MWCNT compared to the CS/Ag system. Based on the results acquired in this study, it is considered propitious to add MWCNT to the nanocomposite for the sake of enhancing the release behavior of 5-FU and the cytotoxicity.

Efficient and safe platforms for the delivery of docetaxel (DTX) for lung cancer therapy produced with chitosan-folate (CS-FA) conjugated MWCNT were developed by Singh, et al. [342]. This material showed up to 79% efficiency of the encapsulation of the drug. The nanosize structure of MWCNT between 230 and 483 nm in length was verified from particle size studies and TEM analysis. An MWCNT sustained release of drugs was revealed from in vitro drug release profiles and the evaluation of cytotoxicity allowed to assess the result of the intracellular content of DTX owing to the increased cell uptake of MWCNT. Compared to Docel^TM^, after twenty-four hours of incubation with A549 cells, DTX-CS-FA-CNAC (CNAC being a conversion of carboxylated MWCNT (CNA) into acylated MWCNT) achieved a reduction of up to 89 times in the IC_50_ value. DTXCHI-FA-CNAC evidenced increased cytotoxicity and low toxicity when correlated with DTX-CNAC, DTX-CS-CNAC, and Docel^TM^, promoting better efficiency and safety, which indicates the possibility of exploiting DTX-CS-FA-CNAC as an effective and safe platform for lung cancer therapy targeting.

Due to doxorubicin’s (DOX) effectiveness in combating various types of cancers, including ovarian and breast cancer, acute leukemia, lung carcinoma, and various sarcomas, this anthracycline cytostatic antibiotic is widely used in cancer chemotherapy [343]. However, due to its brief plasma half-life, cardiac toxicity, and pristine doxorubicin build-up in places other than tumor tissues, its clinical use is restricted [344,345]. A different method for the controlled release of DOX to curb tumor increase and reduce side effects such as its toxicity is drug targeted delivery through polymeric materials [213,346,347,348]. Several natural and synthetic polymers have been utilized to generate sustained drug delivery systems (DDSs), and chitosan is one of them [349,350].

Due to graphene’s unique physical and chemical properties, this nanoparticle has attracted attention in the DDS field. Graphene oxide (GO), a graphene derivative, presents exceptional aqueous solubility, biocompatibility, and multi-functionalities, essential features for delivering anticancer drugs. Therefore, many attempts have been made to load anticancer drugs onto GO efficiently by either non-covalent or covalent bonding [351,352,353].

GO can be a target to DOX through π–π interactions to regulate the DOX release. Besides, the epoxy and carboxylic functional groups present in GO can interact with amine groups present in chitosan (CS), and considering the advantages of each of these elements, the CS/GO nanocomposite consists of an efficacious formulation for controlled release of anti-cancer medication (DOX) [354,355].

CS nanofibers can be produced by blending with polymers such as polyethylene oxide (PEO), aiming to ameliorate stability and mechanical properties. In this context, nanofibrous scaffolds of PEO/CS/graphene oxide (GO) produced by electrospinning for controlled release of doxorubicin (DOX) were developed in the work of Ardeshirzadeh, et al. [356]. The π–π stacking interaction among DOX and GO with thin pores of nanofiber scaffolds demonstrated a more significant drug load (98%) and controlled release of DOX carried in these nanofibers. A strong pH dependence was evidenced by the DOX release results from nanofibrous scaffolds at pH 5.3 and 7.4. Instability can be observed under acidic conditions of the hydrogen bonding interaction between GO and DOX, resulting in an accelerated drug release at pH 5.3. The results of cell viability evidenced that the PEO/CS/GO/DOX nanofibrous scaffold could serve as an option source to prevent the side effects of free DOX and could be utilized as a novel formulation for lung cancer treatment.

Chitosan (CS)-functionalized GO for the controlled release of 5-fluorouracil and ibuprofen was manufactured in the study of Rana, et al. [357]. The authors concluded that graphene sheets (that consist of chitosan-functionalized graphene oxides (FGOCs)) are promising materials for biomedical applications due to long-term biocompatibility and controlled release behavior. The water-insoluble anticancer drug camptothecin (CPT) was loaded into the CS/GO composite for the treatment of HepG2 and HeLa cancer cells in the work of Bao, et al. [358]. The results demonstrated a higher load capacity of GO-CS for CPT and a considerably elevated cytotoxicity in the HepG2 and HeLa cell lines exhibited by the GO-CS-CPT complexes when correlated to the pure drug. GO-CS is additionally capable of condensing plasmid DNA into stable nanosized complexes. The resultant GO-CS/pDNA nanoparticles demonstrate moderate transfection efficiency (which refers to determining the percentage of cells that are transfected—that expresses the desired gene(s) of interest—compared to the entire cell population) in HeLa cells in specific proportions of nitrogen/phosphate. Hence, the authors vouched for the GO-CS nanocarrier’s ability to load and deliver anti-cancer genes and drugs.

#### 4.1.2. Photodynamic Therapy (PDT)

Photodynamic therapy (PDT) of tumors is one of the most active research fields in indirect cancer phototherapy, combining a light source and the local or systemic administration of a photosensitizing agent, which is preferably located within the tumor, for local treatment designed to destroy abnormal tissue lesions, such as cancer [359]. The photosensitizing agent activated by light transfers energy to molecular oxygen to generate reactive oxygen species (ROS), causing the death of the target cell, and once compared with conventional cancer therapy, such as surgery, radiotherapy, and chemotherapy, PDT emerges as a promising non-invasive treatment for cancer [360,361,362,363,364].

Hyperthermia consists of an anti-cancer treatment in which an exogenous energy source is used to promote the healing of the tumors since this heat can increase the therapeutic window of radiotherapy and/or chemotherapy by sensitizing the tumors to these therapies, in addition to the possibility of directly killing cancer cells [365,366,367].

The thermic effect, the cavitation effect, and the non-thermal/non-cavitation effect are the three main mechanisms that influence magnetic fields’ results on tumors/cancer cells [276]. Pulsed magnetic fields (PMFs) were utilized to activate localized hyperthermia in the tissues in which accumulations of magnetic nanoparticles (MNPs) were present [364]. Due to the intolerance to chemotherapy and decreased functionality of several organs presented by advanced cancer patients (stages 3 and 4), treatment with PMF has been suggested for these cases [365].

According to Sengupta and Balla [276], various magnetic fields can influence cancer cell activities. An expressive inhibitory effect was observed in the growth of cancer cells. The production of free radicals in the mold of reactive oxygen species (ROS)/reactive nitrogen species (RNS) occurs through static magnetic fields and induces cancer cell apoptosis. The alternating magnetic fields generate hyperthermia, which, in turn, increases the effectiveness of anti-cancer treatment by facilitating the internalization of drugs and inhibits the proliferation of cancer cells. Owing to the reasonably great understanding of the particular frequencies of tumors, pulsed electromagnetic field (PEMF) treatment seems to have a high potential for application among the different cancer therapies assisted by the magnetic field. Despite the limitation of reports addressing PEMF treatment’s effectiveness in humans, the successful application of PEMF for cancer treatment can be achieved by further exploring these tumor-specific frequencies.

Among the agents used in PDT, ZnO is of particular interest due to its unique optical and electronic properties, widely used for device applications, including transducers and varistors [366]. ZnO nanoparticles can absorb a larger fraction of the UV zone, acting as a photosensitizer. With UV illumination, the ZnO photocatalyst in an aqueous solution can generate ROS, such as hydroxyl radical, hydrogen peroxide, and superoxide, enabling the application of ZnO nanoparticles in the highly efficient decomposition of some organic compounds and induced cytotoxicity against some mutated cells [367,368,369].

In the context of the combined application of ZnO nanoparticles with an anti-cancer drug in PDT, Guo et al. [370] combined the high reactivity of ZnO nanoparticles with daunorubicin, one of the most important anticancer drugs in the clinical treatment of acute leukemia and solid tumors, to inhibit the MDR of drug-resistant leukemia K562/A02 cells. The results showed that, in the presence of ZnO nanoparticles of different sizes, there was an increased cell uptake of daunorubicin for both leukemia cell lines, suggesting that the co-administration of ZnO nanoparticles and the chemotherapeutic drug daunorubicin resulted in synergistic cytotoxic effects in leukemic cancer cells, which has been further enhanced by UV irradiation.

According to the PDT concept of self-illumination, the photoactivation of ZnO nanoparticles is expected to cause higher ROS release levels, which, once correctly targeted to cancer cells, promote their selective destruction. ZnO nanoparticles conjugated to porphyrin (ZnO-MTAP) were synthesized and evaluated for photodynamic therapy (PDT) against ovarian cancer in the work of Zhang et al. [371]. The generation of reactive oxygen species (ROS) was observed in a manner dependent on the conjugates’ concentration and lighting after exposure to ultraviolet light A (365 nm). Additionally, 3 to 6 h after ultraviolet irradiation, the activity of caspase 3/7 was notably high, suggesting that phototherapy was able to induce apoptosis, which indicates that the photoactivation of ZnO-MTAP conjugates may be useful for induction of toxicity in ovarian cancer on exposure to UV-A light, leading to targeted destruction of tumor cells. Therefore, it is concluded from the mentioned studies that the photoactivation of ZnO nanoparticles conjugated with tumor ligands can be useful for the targeted destruction of cancer cells [372].

The potential therapeutic applications of zinc quantum dots have been suggested by photophysical studies based on their ability to absorb energy in the near-infrared region, what indicates the usefulness of Zn in a targeted delivery system, being capable of destroying the defective cells while maintaining the integrity of healthy cells [373]. In this context, nanoparticles constituted of Folic acid (FA) conjugated carboxymethyl chitosan coordinated to manganese doped zinc sulfide quantum dot (FA–CMC–ZnS:Mn) were developed by a simple aqueous route in the work of Mathew et al. [374]. These nanoparticles can effectively be used for targeted drug delivery and imaging the drug carrier’s path by the fluorescence of ZnS:Mn attached to the system, without affecting their metabolic activity and morphology. The size range of 5-Fluorouracil encapsulated FA–CMC–ZnS:Mn nanoparticles were from 130 to 150 nm and the drug encapsulation efficiency obtained was 92.08% with CMC to 5-FU ratio 2:1. In vitro drug release studies showed controlled release of 5-FU from FA–CMC–ZnS:Mn nanoparticles. The FA–CMC–ZnS:Mn nanoparticles were found to be nontoxic to mouse fibroblast L929 cells, but 5-FU encapsulated nanoparticles were found to be toxic to breast cancer cell line MCF-7.

An approach of combining quantum dots (QDs) technology with anti-cancer drug therapy was described in the work of Yuan et al. [375]. The synthesis of blue-light emitting ZnO QDs combined with biodegradable chitosan (N-acetylglucosamine) occurred by a chemical hydrolysis method and exhibited a strong blue emission at 440 nm for tumor-targeted drug delivery. Chitosan enhanced the stability of the QDs because of the hydrophilicity and cationic charge characteristics of chitosan. A water-dispersed ZnO-QD–chitosan–folate carrier was loaded with an anti-cancer drug (DOX), and the drug-loading efficiency was 75%. The drug release response of DOX-loaded ZnO-QD–chitosan–folate carrier was characterized by an initial rapid drug release followed by a controlled release. The results indicate that the proposed QDs loaded with anti-cancer agents and encapsulated with biocompatible polymer represent a potential platform to deliver tumor-targeted drugs.

The introduction of magnetic nanoparticles in the field of cancer therapy takes place in such a way as to optimize the accumulation of the dose of the drug in the tumor interstice through a magnetic gradient, which allows the chemotherapeutic agent to have improved anticancer efficacy and toxicity negligible systemic [376].

Superparamagnetic iron oxide nanoparticles have a strong magnetism and low toxicity, therefore being used as a heating source in the hyperthermia of magnetic fluids. These nanoparticles aggregate at the tumor site and then are heated in an alternating magnetic field [377]. Once started in this field, these nanoparticles transform the energy into heat, becoming powerful heat sources. Besides, magnetic fields may be administered to profound regions of the living body as they are not assimilated by living tissues [378].

Owing to a single magnetic dominion that reveals biocompatibility, easy synthesis, superparamagnetism, non-toxicity, and high magnetization, magnetite (Fe_3_O_4_) is the most widely used magnetic nanoparticle, being convenient for biomedical applications in the areas of magnetic hyperthermia, contrast agents for magnetic resonance, agents for magnetic separation and carriers for drug delivery [379,380,381,382]. Thus, the incorporation of iron oxide particles, such as the magnetite, Fe_3_O_4_, into chitosan (CS) can be done to acquire magnetic CS materials, which may be used in biomedical applications encompassing magnetic fields. The magnetic hyperthermia of chitosan-coated magnetic nanoparticles was investigated, and the systems proved to be biocompatible, in addition to having a particular affinity with KB cells, a human squamous cell carcinoma line derived from the oral floor [383]. Blending [384], polymer microgel template [385,386], and co-precipitation methods [387] are the approaches used for the synthesis of hybrid CS ferrogels. The nanoparticles obtained from the synthesis of magnetite nanoparticles by co-precipitation of iron chlorides in the attendance of CS indicated a typical superparamagnetic behavior [388].

Magnetite nanoparticles (Fe_3_O_4_) were prepared by co-precipitation of Fe^3+^ and Fe^2+^ with an aqueous solution of NaOH in the work of Zhao et al. [389]. Subsequently, magnetic nanoparticles composed of Fe_3_O_4_-chitosan with a core-shell structure with a 30–50 nm diameter were synthesized in two stages using a suspension crosslinking method. The property of inductive heating of these composite nanoparticles in an alternating current (AC) magnetic field was investigated, and their potential for localized treatment for cancer hyperthermia was evaluated. The saturation magnetization (Ms) and coercivity (Hc) were 65.53 emu g^−1^ and 0 Oe for nanoparticles of Fe_3_O_4_ and 24.67 emu g^−1^ and 21.69 Oe for nanoparticles composed of Fe_3_O_4_-chitosan, respectively. After 29 min exposure in the CA magnetic field, the temperatures of the physiological saline suspensions containing Fe_3_O_4_ nanoparticles or nanoparticles composed of Fe_3_O_4_-chitosan were 97.5 °C and 53.7 °C, respectively. Therefore, it is concluded the possibility of using Fe_3_O_4_-chitosan composite nanoparticles to treat localized hyperthermia of cancers.

A multifunctional nano-drug system containing Fe_3_O_4_, O-carboxymethyl chitosan (OCMCs), and curcumin (Cur) was prepared by ex-situ grafting in the work of Thu et al. [390]. The fluorescent staining experiments showed that this system influenced the cell internalization capacity of curcumin and conducted the drug to HT29 cells, as expected, showing the possibility of using this system to monitor the drug’s circulation using the fluorescence technique. From the real-time cell analysis (RTCA), the effect of Fe_3_O_4_/OCMCs/Cur on this cancer cell line was considered much stronger than that presented by pure curcumin. Therefore, this system can be considered an intelligent nanomaterial for drug administration, and since it contains magnetic particles, it can also be considered for hyperthermia therapy for cancer treatment.

A nanodevice consisting of magnetite/chitosan nanocomposites (core/shell) with a magnetic response suitable for intravenous drug administration was designed in the work of Arias et al. [376]. The results showed complete polymeric coverage of the magnetite nuclei. The gemcitabine entrapment procedure in these Fe_3_O_4_/chitosan nanocomposites resulted in a higher drug load and slower drug release properties than the surface adsorption procedure. Additionally, Fe_3_O_4_/chitosan NPs exhibited a pH-responsive release of gemcitabine, a consequence of specific polymer dissolution in acidic environments. An in vivo proof of concept using Prussian blue coloring further confirmed the magnetic targeting capabilities of this magnetite/chitosan nanodevice. The authors concluded that these magnetic NPs could be applied in the treatment of cancer since they are capable of providing appropriate and localized amounts of the anticancer drug gemcitabine in the tumor, and after exposure to an alternating magnetic gradient, cause a selective increase in temperature in the compromised tissue with cancer cells, allowing their apoptosis.

Many properties of biodegradable electrospun CS nanofibers, including Fe_3_O_4_ and Fe^2+^ magnetic nanoparticles made by distinct methods, were explored in the work of Lin, et al. [391], with the measurement of their magnetic characteristics. Electrospun chitosan nanofibers containing magnetic nanoparticles proved to be non-cytotoxic according to in vitro cell incubation experiments, and by applying a magnetic field, they would be able to enhance the temperature of the solution to 45 °C and effectively reduce the rate of proliferation/growth of tumor cells, according to heating experiments. The authors concluded an excellent potential for endoscopic or surgical delivery for hyperthermic cancer therapy of these magnetic chitosan nanofiber composites.

N-((2-hydroxy-3-trimethylammonium) propyl) chitosan (HTCC)/Fe_3_O_4_ magnetic alginate encapsulated (HTCC-MNPs) chlorides were developed in the work of Li et al. [392] using an ionic gelation method and applied to MDR gastric cancer in vivo and in vitro. HTCC-MNPs exhibited good water solubility, efficient cell penetration, and good biocompatibility and significantly reduced cell viability in the SGC7901/ADR drug-resistant cancer cell line. HTCC-MNPs significantly inhibited the growth of gastric MDR tumors. They reduced tumor volume by inducing autophagy and cell apoptosis, attributed to mitochondrial dysfunction and excessive ROS accumulation, suggesting that these HTCC-MNPs may be useful as therapeutic agents in the treatment of gastric cancer by MDR, reversing chemosensitization.

Nanoparticles of superparamagnetic iron oxide functionalized with chitosan (Fe_3_O_4_-CS) and conjugated with the anti-cancer drug doxorubicin (DOX) were synthesized in situ by a simple method of hydrolysis at room temperature in the work of Mahesh et al. [393]. DOX was conjugated to the Fe_3_O_4_-CS nanoparticles through the imine bonded glutaraldehyde crosslinker (−C=N−), sensitive to cleavage around pH 4.4–6.4, pH of the intracellular components of the endosomes/lysosomes in the cell carcinogenic, and the amounts of chitosan used were able to control the crystallite size, dispersibility, suspension stability, and magnetization. In vitro drug release experiments (cell viability) in human breast cancer (MCF7) and ovarian cancer (SKOV3) cell lines indicated that DOX conjugated to magnetic nanoparticles had an enhanced therapeutic effect when compared to the corresponding concentrations of the free drug, and cell toxicity assays in MCF7 cells indicated that pH-sensitive Fe_3_O_4_-Dox nanoparticles have more significant anticancer activity with 1.4 µM IC50 than free Dox with 4.8 µM IC50, with the added advantage of reduced toxicity to normal cells due to the ability to segment.

Chitosan can be combined with magnetic materials such as iron oxide [391], multi-walled carbon nanotubes (MWCNTs) [394], and graphene oxide (GO) [356] in such a way that, in the attendance of the magnetic field, the target of these magnetic materials to tumor sites can be done, increasing the accumulation of anticancer drugs in the target region, and consequently, enhancing the effectiveness of the treatment of drug delivery agents for cancer treatment.

The biological applications of the graphene derivative, graphene oxide (GO), have attracted attention for cell growth and differentiation [395], delivery of genes and drugs [354], and photothermal therapy (PTT), which consists of generating heat from the utilization of red or near-infrared laser (NIR) radiation to treat diseases, substantial tumors treatment [396,397]. Simultaneous activation of toll-like receptor (TLR)-4 and 9 responses of the same autophagy in macrophages and the CT26 colon cancer cell can occur through the use of GO [395], in addition to the injection of this material stimulates the infiltration of immune cells into the tumor bed, curbing the augmentation of colon cancer in mice [398]. Additionally, the death of MCF-7 cells resistant to DOX and CP can be enhanced by the use of GO conjugated with chemotherapeutic drugs (DOX and CP-11) [354].

Outstanding photothermal conversions and the possibility of loading drugs for multimodal therapy are presented by carbon-based nanomaterials, particularly graphene-based nanomaterials, such as reduced graphene oxide nanosheets [399,400], carbon nanotubes (CNTs) [401,402], and quantum dots of graphene [403,404]. Thus, a broad study of these nanomaterials for cancer PTT has been carried out, being the graphitic carbon nanocage (GCNC) a relevant member of the graphene-based nanomaterials family with an internal cavity and can be utilized for a load of drugs [405].

Thus, GCNCs are unequaled graphene-based nanomaterials, which can be applied for photothermal cancer therapy (PTT), even though microwave irradiation assisted PTT of GCNC-based organic/inorganic hybrid biomaterials with low toxicity has not been extensively reported. In this context, chitosan (CS)-coated GCNCs were developed in the work of Li, et al. [406], and the results witnessed a rapid conversion of an 808 nm laser light energy into warmth from GCNCs and GCNCs/CS in mouse tumors, which promotes efficient death of nasopharyngeal carcinoma cells and inhibits tumor growth. The phototoxicity of GCNCs/CS after being carried with 5-fluorouracil (5FU) is increased, damaging tumors more significantly, which were not found after six days of treatment with 5FU-GCNCs/CS under irradiation, what can be attributed to the synergistic effect of the PTT response of GCNCs and 5FU chemotherapy.

CS-GCNCs loaded with 5Fu were also studied by Guo, et al. [407] for its use in cancer therapy once activated by 808 nm laser and microwave co-irradiation. Based on the results, the authors reported that, after coating with CS, there was a significant reduction in the cytotoxicity of GCNCs, cell cycle defects, and in the 5Fu release rate of CS-GCNCs, implying in a sustained release, which could be speed up by 808 nm laser and microwave co-irradiation. It was also observed an enhancement in the level of caspase-3 expression due to the high retention in cancer cell killing bioactivity by the 5Fu in CS-GCNCs, which implies a higher apoptosis-related cysteine peptidase. Thus, an elevated bioactivity was exhibited by 5Fu, and after incorporation into CS-GCNCs, it promotes damage to cancer cells.

Due to the photocatalytic properties, great stability, excellent biocompatibility, and low toxicity presented by TiO_2_, this material’s biomedical applications arouse great concern in experimental and theoretical studies. The advances and medical applications of TiO_2_ in photodynamic therapy (PDT)—which is utilized in drug delivery, cancer treatment, cell imaging, biosensors for biological assays, and genetic engineering, attract considerable attention [408].

Due to the previously mentioned properties presented by TiO_2_ nanoparticles (NPs), they have been widely used in cancer therapy [409,410,411,412]. The photoexcited TiO_2_ demonstrates a useful ability to kill cancer cells due to its excellent photocatalytic activity, which in addition to its low toxicity and excellent photostability allows the application as a photosensitizer for cancer treatment, also being able to be applied in a vital sector for genetic engineering such as nucleic acid endonuclease. The creation of photo-induced electrons and holes can occur when lighting TiO_2_ NPs by UV light with a wavelength less than 385 nm, and these electrons and holes can form powerful oxidative radicals (e.g., OH, HO_2_) from the reaction with hydroxyl ions or water, being these radicals able to destroy the structure of bacteria, fungi and tumor cells [408].

Considering the reduction in toxicity of drug-loaded CS promoted by the GO integration in the polymeric system, TiO_2_/DOX particles were synthesized by Samadi, et al. [413] and scattered in carbon layers of GO leaves. The incorporation of the TiO_2_/DOX/GO synthesized composite into PLA/CS nanofibers allowed the generated nanofibers to be utilized within 2 weeks for the controlled release of DOX for lung cancer treatment. The average fiber diameter of the PLA/CS composite was 170 nm and 140nm for PLA/CS/TiO_2_/DOX/GO, and the loading efficiency was 97%, 94%, and 92% for nanofibers carrying 10, 25, and 50 mg, respectively. The results indicated a lower DOX release rate due to the improvement in PLA/CS content, TiO_2_, and GO/TiO_2_/DOX ratio. With the increment of DOX ratio in the CS/PLA/TiO_2_/DOX/GO nanofibers, there was an increase in the multiplication curbing effect on cancer cells targeting as indicated in the cell viability study. The use of CS/PLA/TiO_2_/DOX/GO nanofibers in an external magnetic field’s attendance implied increased killing of the A549 cancer cell.

Table 3 exhibits a compilation of several studies over the last 10 years on chitosan uses in cancer treatment and each work’s relevant results. It can be seen that this biopolymer has anti-tumor activity, being able to be associated with other polymers or metal NPs as a strategy to increase these polymer characteristics, and once associated with anti-cancer drugs and photothermal therapy, can effectively reduce the proliferation of tumor cells and contribute to accelerated the shrinking and disappearance of tumors.

## 5. Conclusions

The remarkable properties presented by chitosan have aroused the interest of applying this material in several biomedical applications, such as tissue engineering, wound dressing, drug delivery, and cancer treatment.

Chitosan’s biocompatibility and biodegradability explain the use of this polymer in the pharmaceutical field, and its chemical groups support many modifications, which made this polymer an option for use as a controlled drug release agent. Chitosan can act as a vehicle and improve the action of some drugs used against pain, increasing the bioavailability and dissolution properties of low solubility drugs. This polymer can also be combined with other polymers or inorganic materials to modulate its mechanical and chemical degradation properties, a trend observed in the studies over the last two decades.

Since chitosan is easily soluble in dilute acidic solutions and is degraded in vertebrates by lysozyme and bacterial enzymes in the colon, this polymer is an exceptional carrier for anticancer drugs. Chitosan has inhibitory effects on tumor cell growth, tumor-induced angiogenesis, and tumor metastasis, thereby showing good anticancer activity. Chitosan-based systems can be used for the encapsulation and targeted delivery of antineoplastic drugs such as cisplatin, 5-FU, and DOX, providing a longer drug retention time, and a reduction in the administration frequency, in addition to a decrease in the systemic toxicity—thereby achieving a safer drug delivery to tumors.

Chitosan is also applied in tissue regeneration systems, with the ability to form extremely porous scaffolds structures with interconnected pores and intrinsic antibacterial activity that allows cells to be seeded, migrate into the inside, multiply, and be provided by enough nutrients. Chitosan polar groups provide a microenvironment with favorable physicochemical properties for cell adhesion and proliferation.

Chitosan scaffolds provide support for the fixation and multiplication of osteoblast cells, and also the generation of mineralized bone matrix. Chitosan-based hydrogels stand out for the regeneration of cartilaginous tissue, efficiently supporting chondrogenic activity. Chitosan-based materials have also been shown to promote adhesion, survival, and neurite outgrowths of neurons; chitosan’s potential in nerve regeneration has been demonstrated in vitro and in vivo.

Concerning wound healing, chitosan-based hydrogels are biocompatible and can supply a humid wound environment, protect against secondary infections, eliminate wound exudate, produce accelerated healing, and lead to soft scars. Besides, this material may either act as an obstacle to prevent the invasion and proliferation of microorganisms, or contain the infection, and supply structures for cell growth and proliferation. Additionally, since chitosan is a hemostat, it supports natural blood coagulation and acts as a barrier for nerve endings, thereby decreasing pain.

The demand for natural biomaterials is continuous and growing, as they consist of a class of materials extracted from renewable sources with high availability. In the last few years, studies using chitosan in the areas of biomedicine addressed throughout this review have been intensified due to the outstanding results observed. This has caused chitosan to be considered one of the most important and promising polymers for biomaterials applications. The future perspectives for chitosan in biomaterials involve an intensification of its commercial use with the development of more reproducible and less costly techniques for its production and the evolution of surface modification treatments that will improve its use.

## Figures and Tables

**Table 1:** Studies of chitosan (CS) uses in tissue engineering and wound healing over the last 10 years.

System	Field of Study	Application	Relevant Results	Reference
CS film	Wound healing	CS film on wound repair in distal horse limb	CS membrane accentuated the formation of granulation tissue, emphasizing a potential repairing effect	Martins et al., 2013 [191]
CS/PVA hydrogel nanofiber mats	Wound healing	Nanofiber mats and hydrogel application in wound healing	The nanofiber mats showed good bactericidal activity against *Escherichia coli*, combining the advantages of nanofibrous mats and hydrogel dressing	Liu et al., 2014 [213]
CS–Alg and CS-xanthan polyelectrolyte complexes incorporated with erythromycin	Wound healing	CS membranes complexed with alginate or xanthan for the treatment of skin lesions	The release of the erythromycin molecule from the matrices was slow, requiring less frequent changes and resulting in less traumatic and more comfortable treatment for the patient	Souza et al., 2014 [212]
CS	Wound healing	Healing effect of low molar mass and degree of acetylation chitosan on skin lesions of rats	CS plays an important role in the recovery of acute skin lesions in rats, accelerating the healing process and providing a reduction in the width of the lesion	Fráguas et al., 2015 [204]
CS/gelatin /PVA-based hydrogels	Wound healing	Hydrogels for wound dressing applications	Hydrogels analyzed rapidly absorbed blood from the damaged tissue and adhered to the dermal surface, blocking broken blood vessels, and stimulating platelet release	Fan et al., 2016 [211]
CS-Ag hydrogel	Wound healing	Novel chitosan hydrogels reinforced with silver NPs for accelerating wound healing	The hydrogel exhibited a porous 3D network and ultrahigh mechanical properties due to the intermolecular and intramolecular interactions. Ag NPs integrated into CS hydrogel enhanced the antibacterial properties and also reinforced its mechanical properties	Xie et al., 2018 [214]
CS/PEO/silica hybrid nanofibers	Bone tissue engineering	Hybrid nanofibers as a potential biomaterial for bone repair and regeneration	Proved to be cytocompatible, promoting the attachment and proliferation of murine osteoblast-like 7F2-cells, and also enhance a fast apatite formation by incorporation of Ca^2+^ ions and subsequent immersion in modified simulated body fluid (m-SBF)	Toskas et al., 2013 [100]
CS–Alg and CS–Alg–fucoidan scaffolds	Bone tissue engineering	Biocomposite containing fucoidan for bone tissue engineering	In vitro studies using the MG-63 cell line revealed a good cytocompatibility, increased alkaline phosphatase secretion, and enhanced cell proliferation in the CS–Alg–fucoidan scaffold, and two times higher protein adsorption and mineralization in comparison with CS–Alg scaffold	Venkatesan et al., 2014 [117]
CS/ Hydroxyapatite(HA)/gelatin scaffolds	Bone tissue engineering	Scaffolds obtained by aggregation of CS/ HA spheres for bone tissue regeneration	A 3D structure was formed, and the HA acts as reinforcement until the concentration of 3%	Oliveira et al., 2015 [104]
HA/gelatin–CS core-shell nanofibers	Bone tissue engineering	Biomimetic composite scaffolds for bone tissue engineering	Gelatin-CS core-shell structured nanofibers improved HA’s mineralization efficiency and formed a homogeneous HAP deposit compared with CS and CS-gelatin nanofibers. When Human osteoblast-like cell line (MG-63) were cultured on the materials, the results showed that HA deposited on the Gelatin-CS core-shell nanofibers could further enhance osteoblast cell proliferation	Chen et al., 2018 [103]
Apatite-CS scaffold	Bone tissue engineering	A composite scaffold as a potential injectable construct for bone tissue engineering	The scaffold presented an osteogenic-like microenvironment adequate for biomineralization. MC3T3 preosteoblast cells differentiation and subsequent biomineralization, revealed by ALP enzymatic activities over seven days of culture and in vitro studies, showed good morphology and cell adhesion	Jahan et al., 2018 [108]
Cellulose nanocrystals (CNC) on CS–Alg-HA scaffolds	Bone tissue engineering	Composite scaffold for bone tissue engineering	Scaffolds containing CNC exhibited an enhancement in swelling, porosity, and compression strength. The results also evoked that the scaffold comprising CNC has a promising cell growth and cell adherence	Shaheen et al., 2018 [118]
Microporous methacrylated glycol CS montmorillonite nanocomposite	Bone tissue engineering	Nanocomposite hydrogel for bone tissue engineering	The hydrogels formed an interconnected microporous network and promoted the proliferation and the attachment and induced the differentiation of encapsulated mesenchymal stem cells in vitro	Cui et al., 2019 [106]
Composite scaffolds of Cu–CS derivative and a Sr-substituted HA	Bone tissue engineering	Material platforms containing therapeutic ions for bone tissue engineering	While Sr is uniformly released to assist the regeneration process for an extensive time, Cu has an explosive release from CS, enable possible bacterial colonization to be quickly stopped	Gritsch et al., 2019 [107]
Genipin-crosslinked and fucoidan (FD)-adsorbed nano-hydroxyapatite/hydroxypropyl chitosan	Bone tissue engineering	Composite scaffolds for bone tissue engineering	The scaffolds showed an open structure with interconnected pores and a rough morphology. The incorporation of n-HA and adsorption of FD increased ALP activity in 7F2 osteoblast cells and promoted their mineralization	Lu et al., 2019 [109]
CS/carboxymethyl cellulose reinforced with multiphasic calcium phosphate	Bone tissue engineering	Whisker-like fibers for bone tissue engineering	The composite scaffolds exhibited desirable microstructures with high porosity and interconnected pores. Scaffolds presented an improved in vitro proliferation, attachment, and mineralization of MG63 cells, and triphasic fibers were more effective in reinforcing the scaffolds and resulted in higher cell viability	Matinfar et al., 2019 [110]
Novel freeze-dried silanated CS scaffold reinforced with HA nanofiber	Bone tissue engineering	Novel freeze-dried scaffold for bone tissue engineering	CGH25 scaffold (15% of GPTMS and 25% of n-Has) presented better mechanical properties and exhibited a uniform distribution of n-HAs with an appropriate porous microstructure, porosity percentage, and water uptake. It was also observed a proper biomineralization behavior after 14 days of soaking in SBF solution and the viability of MG-63 osteoblastic cells improved concerning chitosan scaffold for up to 72 h of cell culture	Nezafati et al., 2019 [111]
Poly (3-hydroxybutyrate)- CS/alumina nanowires	Bone tissue engineering	Composite scaffold for bone tissue engineering applications	A formation of calcium phosphate sediments on the surface of alumina containing scaffolds after 7 and 28 days of immersion in SBF was observed. Proliferation and viability of MG-63 cells and alkaline phosphatase secretion are significantly higher on scaffolds containing alumina than that of the PHB or PHB-CS	Toloue et al., 2019 [119]
CS-based hydrogel	Bone tissue engineering	Injectable hydrogel for bone tissue engineering	It was observed on the surface and in the hydrogel’s cross-section, an interconnected porous structure suitable for transporting extracellular fluid, nutrients, and hormones to cells and waste removal. Moreover, the SaOS-2 cells adhered and proliferated well on the hydrogel	Saekhor et al., 2019 [120]
Electrospun poly(hydroxybutyrate)/chitosan fibrous scaffolds	Cartilage tissue engineering	Composite scaffold for cartilage tissue engineering	The results evidenced an increase in the hydrophilicity and the mass loss percentage, and the scaffolds’ rate with CS. They maintained the mechanical properties in an appropriate range for the cartilage tissue engineering application. Additionally, in PHB/CS scaffolds, the chondrocytes attached well, spread, and slightly penetrated the polymer matrix of the fibers	Sadeghi et al., 2016 [152]
Chitosan/Poly(vinyl alcohol) (PVA)/graphene oxide (GO) composite nanofibers	Cartilage tissue engineering	Composite nanofibers for cartilage tissue engineering	The addition of GO increased the mechanical properties of the nanofibers. A cell proliferation assay was performed after day 14, and the results showed that the chitosan/PVA/GO (6% by weight) composition provides the most suitable environment for the growth of ATDC5 cells	Cao et al., 2017 [153]
A thermoresponsive chitosan-g-poly(N-isopropylacrylamide) (CS-g-PNIPAAm) copolymer	Cartilage tissue engineering	Thermoresponsive hydrogels for cartilage tissue engineering applications	The microengineering constructions mimicked the shape and organization of the superficial zone of cartilage. After 28 days of incubation in a chondrogenic medium, the mesenchymal stem cells (MSCs) encapsulated in the synthesized hydrogel showed a 6 and 7 fold increase in the secretion of glycosaminoglycans (GAGs) and total collagen.	Mellati et al., 2017 [126]
Hydroxybutyl CS (HBC)/oxidized chondroitin sulfate (OCS) hydrogels	Cartilage tissue engineering	Hydrogels fabricated by 3D bioprinting technique for cartilage tissue engineering	Human adipose-derived mesenchymal stem cells could be 3D cultured in HBC/OCS hydrogel, maintaining good viability. Moreover, the hydrogels were found to trigger the least amount of pro-inflammatory gene expression of macrophage and to inhibit acute immune responses in 7 days	Li et al., 2018 [149]
Photocrosslinkable maleilated chitosan (MCS)/ methacrylated silk fibroin (MSF) micro/nanocomposite hydrogels	Cartilage tissue engineering	Micro/nanocomposite hydrogels as potential scaffolds for cartilage tissue engineering	The hydrogel exhibited the compressive modulus of 0.32 ± 0.07 MPa when the MSF content was 0.1%, a value in the articular cartilage compressive modulus range. The results of in vitro cytotoxic evaluation and cell culture evidenced the biocompatibility of the hydrogels with TGF-β1 to mouse articular chondrocytes and the capacity to support cells attachment well	Zhou et al., 2018 [150]
Graphene oxide (GO)-CS scaffolds	Cartilage tissue engineering	Composite scaffolds for cartilage tissue engineering	As GO concentration increases, it was observed an improvement in the physical and mechanical properties and an augmentation in the proliferation of human articular chondrocytes in the nanocomposite scaffold, especially in extended cultivation periods (14 days). Additionally, it was observed a more spherical morphology of the human articular chondrocyte on the cross-linked scaffolds for 21 days of culture in vitro	Shamekhi et al., 2019 [154]
Thiolated chitosan (CS-NAC) and silk fibroin (SF) hydrogels	Cartilage tissue engineering	Dual network hydrogels for cartilage tissue engineering	Examinations of dry CS-NAC/SF gels revealed a highly porous structure with well-interconnected pores. Cell culture demonstrated that CS-NAC/SF gels supported the growth of chondrocytes while effectively maintaining their phenotype	Liu et al., 2020 [151]
Silk Fibroin-Gelatin-CS composite scaffolds	Tissue engineering applications	Composite scaffold for tissue engineering applications	The addition of silk fibroin to the composites increased the mechanical strength and degradation rate. The composite scaffolds improved endothelial cell attachment and growth compare to silk fibroin alone	Asadpour et al., 2019 [215]
Agarose/CS/GO (ACGO) composite	Tissue engineering applications	Composite scaffold for tissue engineering applications	The ACGO composite scaffolds exhibited the well -defined interconnected pores with rough surface morphology and showed adequate hemocompatibility and Vero cell proliferation ability. Additionally, the GO present in the composite scaffolds provided a favorable environment for cell attachment and proliferation	Sivashankari and Prabaharan 2019 [216]
Cs/gelatin/hyaluronic acid (HA)/heparan sulfate (HS) composite scaffolds	Engineering of neural tissue	Composite scaffold for neural tissue engineering	The presence of HA and HS in the scaffolds promoted a relevant initial NS/PCs adhesion and supported the long-term growth in a 3D environment. Additionally, NS/PCs in the Cs/Gel/HA/HS scaffold maintained multilinear differentiation potentials with increased neuronal differentiation	Guan et al., 2013 [169]
Cell-Seeded Chitosan Films	Engineering of neural tissue	Films for peripheral nerve tissue engineering	Results demonstrate an excellent cytocompatibility of the chitosan substrate, which allowed neurites to grow from dissociated sensory neurons, what is additionally supported in films preseeded with rat Schwann cells (SCs)- rat bone-marrow-derived mesenchymal stromal cells (BMSC) co-cultures	Wrobel et al., 2014 [157]
Chitosan/gelatin-based scaffolds	Engineering of neural tissue	Conductive scaffold for nerve tissue engineering	An increase in the scaffolds’ electrical and mechanical properties was observed with the incorporation of polyaniline/ graphene (PAG) NPs, and a decrease in the porosity and the water retention capacity. Additionally, conductive scaffolds exhibited a relatively slower degradation, and among the PAG- incorporated scaffolds, the largest number of attached Schwann cells was observed in the scaffolds with 2.5 wt.% PAG (C2G6-PAG2.5).	Baniasadi et al., 2015 [177]
Polycaprolactone (PCL)/chitosan (CS) scaffold	Engineering of neural tissue	Composite scaffolds for nerve tissue engineering	The adherence of Schwann cells (SC) to the scaffolds was proven, and the scaffold containing 5 wt.% of CS exhibited a more significant proliferation on days 1 and 3. Additionally, the scaffolds proved to be non-cytotoxic, and when compared to pure PCL or CS scaffolds, the CS/PCL scaffolds showed better cell distribution, and the cells exhibited an SC phenotype	Bolaina-Lorenzo et al., 2016 [179]
CS–aniline oligomer and agarose hydrogel	Engineering of neural tissue	Self-gelling electroactive hydrogel for neural tissue engineering with on-demand drug release	It was observed that the hydrogels’ conductivity was −10^−4^ S/cm, which can mimic the conductivity of the tissue. The aniline oligomer prolonged the release rate of the drug and rendered electroactivity to the hydrogel, increasing cellular activities, such as proliferation. Additionally, the hydrophobic nature of the aniline oligomer repels water and biological fluids, thus decreasing the swelling rate and the degradation rate.	Bagheri et al., 2019 [178]
Poly (ε-caprolactone) (PCL)/CS/PolyPyrrole (PPy) nanofibrous scaffold	Engineering of neural tissue	Composite scaffold for neural tissue engineering	Chitosan promoted a considerable increase in the hydrophilicity of the scaffold and a decrease in the fiber diameter. The combination of the nanofibrous structure with a greater hydrophilicity favors neural growth, cell attachment, and proliferation. In-vitro studies using PC12 cells revealed that the PCL/CS/ PPy scaffold supports cell attachment, spreading and revealed a significant increase in proliferation up to 356% in comparison to Pure PCL and neurite extension of PC12	Sadeghi et al., 2019 [180]
Alg/CS hydrogel containing olfactory ectomesenchymal stem cells (OE-MSCs)	Engineering of neural tissue	Composite hydrogel for sciatic nerve tissue engineering	Results confirmed that the hydrogel could provide a suitable substrate for cell survival. Additionally, Alg/CS hydrogel with OE-MSCs applied to the sciatic nerve defect enhances regeneration compared to the control group and hydrogel without cells.	Salehi et al., 2019 [181]

**Table 2:** Studies over the last ten years about chitosan (CS) uses in drug delivery systems (DDSs).

System	Drug Loaded	Relevant Results	Reference
CS NPs	Tacrine	The particles showed adequate drug-loading capacity and in vitro release studies showed an initial burst release, followed by a continuous and slow release of the drug. Additionally, polysorbate 80 coating of NPs slightly reduced the drug diffusion-controlled release, and followed the Fickian mechanism	Wilson et al., 2010 [259]
CS-cyclodextrin, carboxymethyl-b-cyclodextrin (CM-b-CD) hybrid nanocarrier	Sulindac	The formation of NPs and encapsulation of sulindac was confirmed through DSC and FTIR analysis. Release profiles indicate a continuous release of the drug throughout 24 h. However, the rate of release was faster during the first hours, being about 55–90% of the drug released after 3 h	Ammar et al., 2011 [260]
CS–Alg Polyelectrolyte Complex NPs	Amoxicillin	The release mechanism of Amoxicillin from the CS-ALG PEC NPS was a combination of both diffusion and erosion. The in vitro drug release studies for 6 hrs revealed the PEC system’s gastroprotective nature and diffusion through the swollen polymeric complex as the main drug release mechanism (Higuchi model), which may significantly reduce the chances of incomplete eradication and systemic side effects.	Arora et al., 2011 [261]
CS/NaHCO_3_ system	Dipyridamole (DP)	The release profiles exhibited a fast release rate on the first day, then a gently stable release in 16 days, followed by an accelerating linear release over 30 days. For the gelling systems of three formulations in this study, the chitosan hydrogel from one with moderate NaHCO_3_ concentration at 0.10 mol/L had the lowest DP release rate, which was mainly attributed to its stronger gel structure	Liu et al., 2011 [262]
CS Oligosaccharides (COS)-coated NLC (nanostructured lipid carrier)	Flurbiprofen	The particle was coated with the COS polymer surrounding the surface uniformly, which positively charged NLC dispersions, providing a longer retention time by interacting with the negative mucous. It is also noticed the superior mucoadhesive properties of COS-coated NLCs, beneficial to the ocular drug delivery system.	Luo et al., 2011 [263]
CS–carboxymethyl starch (CMS) NPs	5-aminosalicylic acid (5-ASA)	An initial burst release was observed, which can be attributed to the desorption of loosely attached 5-ASA from the matrix polymers’ surface. The greatest release for 5-ASA-loaded CS–CMS NPs occurred in the release media of high ionic strength, and a significantly small portion of 5-ASA was released in water. Additionally, the amount of drug released from NPs decreased on increasing cross-linking time	Saboktakin et al., 2011 [264]
CS enclosed mesoporous silica NPs (MSN)	Ibuprofen (IBU)	In vitro release properties of samples in a pH range from pH 7.4 to 6.8 was observed, and the release of IBU showed sensitivity to the release medium pH value. The great amount of IBU released was achieved in pH 6.8, pH value around some tumor cells and inflammatory tissues	Chen et al., 2012 [265]
Low molecular weight (LMW) CS NPs	Erythrocytes	LMW CS NPs showed good compatibility with erythrocytes and can be easily attached to the erythrocyte membrane’s surface, suggesting a potential vascular drug delivery system. The composite drug delivery system contains no harmful components and is entirely biodegradable.	Fan et al., 2012 [266]
CS-coated mesoporous silica nanospheres	Ibuprofen (IBU)	A pH-responsive release of IBU has been achieved by varying the shell structure of positively charged CS in the designed pH 4.0–7.4 solution. Under basic conditions, CS forms a gel-like structure that is insoluble and prevents IBU release at pH 7.4. When the pH is below its isoelectric point (6.3), the drug has been released due to protonation of the amino group on CS	Popat et al., 2012 [267]
Ellagic acid encapsulated CS NPs (EA@CS-NP)	Ellagic acid (EA)	The drug-encapsulation and loading-efficiency of the NPs were 94 ± 1.03% and 33 ± 2.15%, respectively. The drug is entirely released in a phosphate buffer medium by 48 h at room temperature, indicating a sustained drug release pattern of EA from EA@CS-NP.	Arulmozhi et al., 2013 [268]
Novel CS-Functionalized Spherical Nanosilica Matrix (CTS-SNM)	Carvedilol (CAR)	The release manner showed a pH dependency, and after the burst release of drug adsorbed on the CTS-SNM surface or mixed with the CS shell, the release pattern of CAR from the CAR-CTS-SNM pores took place in pulses with a rise in pH all along the gastrointestinal tract, regulated by the swelling effect of CS under acidic environment and the shrinking effect in a relatively alkaline environment	Sun et al., 2013 [269]
CS/sodium tripolyphosphate (TPP)-hyaluronic acid-based NPs	Ceftazidime (CFT)	In vitro release and permeation studies showed a prolonged drug release profile from the CS/TPP-hyaluronic acid NPs gel formulation. Furthermore, the gel formulations were not cytotoxic on ARPE-19 and HEK293T cell lines. The formulation was stable, and the drug activity, as shown by microbiological studies	Silva et al., 2017 [270]
CS/montmorillonite films	Ibuprofen (IBU)	Homogeneous systems with good drug dispersion were obtained, and a slower and more controlled release rate of the drug was observed, indicating that the development of this bio-nanocomposite is promising for DDS	Tavares et al., 2017 [235]
CS/hyaluronic acid hydrogel	Vancomycin	Vancomycin-loaded hydrogels were more reliable and elastic, and the gelation time was shortened. Additionally, the delivery of vancomycin was sustainable for 7 d, reducing the abuse of antibiotics, and the releasing rate was dependent on pH levels	Huang et al., 2018 [271]
Alg/CS hydrogel embedded alginate microspheres	Bull Serum Albumin (BSA)	The controlled release of BSA encapsulated within composite hydrogels showed a significantly lower rate when compared with control hydrogel or microspheres alone, what indicates that this hydrogel/microsphere composite system shows excellent potential for a variety of controlled drug delivery applications, especially in protein delivery	Xing et al., 2019 [272]

**Table 3:** Studies of chitosan (CS) and chitosan-based systems’ uses in cancer treatment over the last 10 years.

Material	Application	Relevant Results	Reference
CS/β-glycerophosphate (β-GP) thermo-sensitive gel	Delivery of ellagic acid (EA) for brain cancer treatment	In the anti-tumor activity study, the results showed that the CS/β-GP gel loaded with EA could inhibit cancer cells’ growth in an EA concentration-dependent manner. Additionally, the CS gels containing 1% (*w*/*v*) of EA significantly reduced viability of U87 7 human glioblastoma cells and C6 rat glioma cell line compared with the CS gels without the presence of EA at 3 days incubation	Kim et al., 2010 [414]
CS/GO composite loaded with anticancer drug camptothecin (CPT)	Treatment of HepG2 and HeLa cancer cells	The results demonstrated a higher load capacity of GO-CS for CPT and a considerably elevated cytotoxicity in the HepG2 and HeLa cell lines exhibited by the GO-CS-CPT complexes when correlated to the pure drug. GO-CS is additionally capable of condensing plasmid DNA into stable nanosized complexes, and the resultant GO-CS/pDNA NPs demonstrate moderate transfection efficiency in HeLa cells in specific proportions of nitrogen/phosphate	Bao et al., 2011 [358]
CS–Alg/Cloisite 30B nanocomposites	Delivery of Curcumin for cancer treatment	The drug was released in a controlled manner, with a much more pronounced release in the basic medium than the acidic medium, and this controlled delivery device presents the advantage of using biodegradable polymers, what implies in no need for surgical removal	Malesu et al., 2011 [415]
CS-functionalized graphene oxides (FGOCs)	Investigate the release behavior of 5-fluorouracil and ibuprofen	IBU and 5-FU were loaded successfully on FGOCs sheets, despite lower numbers of π electrons, and it was observed a controlled release behavior and long- term biocompatibility of FGOCs graphene sheets. Additionally, 5-FU loaded graphene sheets (FGOCs/5-FU) induced significant MCF-7 cancer cell death and presented reduced toxicity to non-targeted cells.	Rana et al., 2011 [357]
CS nanocarrier (NC)-based delivery of Ag NPs	Cytotoxic efficiency of the Ag-CS NC system in human colon cancer cells (HT 29) to cancer therapy	The 50% decrease in HT 29 cells’ viability is achieved for an Ag NPs concentration of 0.33 μg mL^−1^. Ag-CS NCs treatment increased the production of intracellular ROS, indicating that the increase in apoptosis induction in HT 29 cells can be obtained by oxidative stress, moreover to the classic caspase signaling pathway	Sanpui et al., 2011 [326]
CS nanofibers including Fe_3_O_4_ and Fe^2+^ magnetic NPs	Hyperthermia treatment of tumor cells	This material proved to be non-cytotoxic according to in vitro cell incubation experiments, and by applying a magnetic field, they would be able to enhance the temperature of the solution to 45 °C and effectively reduce the rate of proliferation/growth of tumor cells	Lin et al., 2012 [391]
Stearic acid–grafted chitosan (CS-SA)	Carrier for a brain-targeting drug delivery system.	DOX was entrapped into CS-SA micelles with a high drug-loading capacity and exhibited slow drug-release behavior, with a cumulative release up to 72% within 48 h in vitro. CS-SA/DOX micelles showed a therapeutic effect on C6 in vitro, and a relatively high amount of DOX was found in the brain, while a lower amount of drug was accumulated in the heart	Xie et al., 2012 [416]
Ellagic acid encapsulated CS NPs (EA@CS-NP)	DDS in a human oral cancer cell line (KB)	EA@CS-NP exhibit significant cytotoxicity in KB cells in a dose-dependent manner with a very low IC50 value compared to the free EA. In vitro anticancer studies showed a significant antiproliferative effect on the human oral cancer KB cell line, suggesting that CS acted as a promising cancer drug carrier that enhanced the anticancer property of the polyphenolic compound EA.	Arulmozhi et al., 2013 [268]
Au/CS/TiO_2_-Gr composite	An amperometric immunobiosensor for the detection of α-fetoprotein	This immunobiosensor was considered a new tactic for AFP detecting with great sensitivity, bioactivity, and selectivity with suitable storage stability	Huang et al., 2013 [316]
CS NPs	Tamoxifen (Tam) carriers for effective anti-tumor activity in breast cancer cells	Tam-loaded CS NPs induced remarkable improvement in anticancer activity, as demonstrated by MTT-assay, AO/EtBr, and Hoechst nuclear staining. In human breast cancer MCF-7 cells, it was demonstrated that Tam-loaded CS NPs increases the intracellular concentration of Tam and enhance its anticancer efficiency by inducing apoptosis in a caspase-dependent manner	Vivek et al., 2013 [417]
CS-modified PLGA NPs	Surface for Improved Drug Delivery of 5-fluorouracil (5-FU)	After introducing chitosan, the zeta potential of the PLGA nanoparticle surface changed from negative charge to positive one, making the drug carriers more affinity to cancer cells. The positive charge and hydrophilic property induced a moderate and prolonged drug release and a high cumulative drug release. CS-modified PLGA NPs showed the versatility of surface and a possible improvement in the efficacy of current PLGA-based DDS	Wang et al., 2013 [418]
Hydroxybutyl chitosan (HBC)	Thermo-sensitive hydrogel to deliver Doxorubicine hydrochloride (DOX-HCl) for cancer treatment	The in vitro study showed that the HBC solution had low cytotoxicity. The DOX-HCl (1 mg/mL) loaded HBC gels displayed slow release rates independent of the HBC concentration and significantly reduced viability of 4T-1 cells compared with the HBC gels after 1-day incubation. The DOX_HCl loaded HBC gel inhibited rat breast cancer cell growth in a DOX_HCl concentration-dependent manner.	Wang et al., 2013 [419]
Thiolated CS (TCS) NPs loaded with curcumin/5-FU	Colon cancer treatment	5-FU-TCS-NPs and CRC-TCS-NPs systems showed an initial burst release at pH 4.5 and 7.4, followed by a sustained pattern. The in vitro studies proved the enhanced anticancer effects (2.5 to 3 fold) of combined treatment compared to the individual and control bare drugs. The in vivo studies proved the improved plasma half-life of nanoentrapped 5-FU and the CRC up to 72 h	Anitha et al., 2014 [420]
CS coated magnetic nanoparticles (CS MNPs)	Target of DOX to the tumor site under a magnetic field	Fluorescence microscopy images have revealed that DOX loaded CS MNPs were taken up by the cells and accumulated around the nucleus so that DOX is successfully delivered near to the nucleus, where the drug shows its anti-cancer activity. Additionally, DOX loaded CS MNPs were more toxic on Doxorubicin resistant MCF-7 cells than the free drug.	Unsoy et al., 2014 [421]
CS NPs	Oxaliplatin drug delivery system to breast cancer cell therapy	A faster release from the DDS was observed at pH 4.5 than at pH 7.4, a desirable characteristic for tumor-targeted drug delivery. It was found that expression of Bax, Bik, cytochrome C, caspase-9, and -3 was significantly up-regulated while the Bcl-2 and Survivin were inhibited in breast cancer MCF-7 cells. These cells cultured with NPs for 48 h became apoptotic in a caspase-dependent manner indicate that CS NPs could act as an efficient DDS importing Oxalipaltin to target cancer cells	Vivek et al., 2014 [422]
Cyclic RGD-modified CS/graphene oxide (GO)	Drug delivery system of Doxorubicin (DOX) for hepatocellular carcinoma-targeted therapy and imaging	Cellular uptake and proliferation studies using hepatoma cells (Bel-7402, SMMC-7721, HepG2) indicated that the cRGD-modified CS/GO could recognize hepatoma cells and promote drug uptake by the cells. The system exhibits a pH-responsive behavior due to the hydrogen bonding interaction between GO and RC, with a slow-release at a lower pH (tumor environment). A higher pH leads to a weaker hydrophobic interaction and hydrogen bonding, resulting in a faster release rate.	Wang et al., 2014 [423]
Nanofibrous scaffolds of PEO/CS/GO	Controlled release of DOX for lung cancer treatment	The π-π stacking interaction among DOX and GO with thin pores of nanofiber scaffolds demonstrated a more significant drug load (98%) and controlled release of DOX. Instability can be observed under acidic conditions of the hydrogen bonding interaction between GO and DOX, resulting in an accelerated drug release at pH 5.3	Ardeshirzadeh et al., 2015 [356]
CS-decorated DOX-encapsulated NP	Target and elimination of tumor reinitiating cancer stem-like cells	CS can eliminate tumor-initiating cancer stem cells by a DOX delivery to the tumor microenvironment. It was observed a 6 times increase in the cytotoxicity of the DOX when compared to the use of free DOX for eliminating CD44^+^ cancer stem-like cells residing in 3D mammary tumor spheroids and a reduction in the size of the tumor in an orthotopic xenograft tumor model with no evident systemic toxicity	Rao et al., 2015 [278]
Nanocomposites of CS/Ag and CS/Ag/MWCNT encapsulated with 5-FU	Study the release profile and cytotoxicity of this system towards an MCF-7 cell line	It was observed the encapsulation of 5-FU and sustained and prolonged release of this drug in the case of the CS/Ag/MWCNT nanocomposite, and more significant cytotoxicity with an IC_50_ of 50 μg/mL when related to the CS/Ag system	Nivethaa et al., 2016 [333]
Graphene oxide (GO)-carboxymethyl chitosan (CMC)-fluorescein isothiocyanate- lactobionic acid (LA)	Targeted anticancer drug delivery systems with DOX	It was observed a high drug loading content and efficiency (>96%) and pH-sensitive release, being faster at the mildly acidic pH typical of the tumor microenvironment and slow and sustained manner at the normal physiological pH. In vitro tests on a liver cancer cell line demonstrated that DOX-loaded systems effectively induce cell death, being almost as potent as the free drug. Further, the DOX-loaded LA conjugated GO system was able to selectively induce the death of cancerous cells and also was non-toxic to a non-cancerous cell line	Pan et al., 2016 [424]
CS/PVA	Potential use in the controlled release of cisplatin	The amount of Cisplatin incorporated in the chitosan/PVA beads was 5mg per mg of beads. Authors concluded that the chitosan/PVA system presented a vehicle behavior for drug release of cisplatin	Souza et al., 2016 [281]
CS-folate (FA) conjugated MWCNT	Efficient and safe platforms in the delivery of docetaxel (DTX) for lung cancer therapy	This material showed an up to 79% efficiency of encapsulation of the drug, and a sustained release of drugs was revealed. Compared to that of Docel^TM^, after 24 h of incubation with A549 cells, DTX-CS-FA-CNAC achieved a reduction of up to 89 times in the IC_50_ value. Additionally, DTXCHI-FA-CNAC evidenced increased cytotoxicity and low toxicity when correlated to DTX-CNAC, DTX-CS-CNAC, and Docel^TM^	Singh et al., 2017 [342]
CS-coated GCNCs	Photothermal cancer therapy (PTT)	The phototoxicity of GCNCs/CS further damages the tumors after loading with 5-FU. Tumors are no longer detected after 6 days of 5FU-GCNCs/CS treatment under irradiation, promoting efficient death of nasopharyngeal carcinoma cells and inhibiting tumor growth	Li et al., 2018 [406]
CS	Enhancement of the anti-tumor activity of natural killer (NK) cells by activating dendritic cells (DC)	They found that DCs are directly activated by CS, what improve the effector functions of human NK cells, promoted the survival of these cells, and enhanced their cytotoxicity averse to leukemia cells, implying in a better antitumor activity in vivo	Li et al., 2018 [11]
CS-grafted-dihydrocaffeic acid and oxidized pullulan hydrogels	pH-responsive injectable hydrogels for localized delivery of Doxorubicin (DOX)	The hydrogels showed good DOX release, effectively killing colon tumor cells (HCT116 cells) and good antibacterial properties against *E. coli* and *S. aureus* in vitro when the antibacterial model drug amoxicillin was encapsulated in the hydrogels, suggesting that this material is a candidate for the development of colon cancer drug delivery carriers	Liang et al., 2018 [425]
PLA/CS/TiO_2_/DOX/GO	Controlled release of DOX for lung cancer treatment	The results indicated a lower DOX release rate due to the improvement in PLA/CS content, TiO_2,_ and GO/TiO_2_/DOX ratio. With the increment of the DOX ratio, there was an increase in the multiplication curbing effect on cancer cell targeting. The use of this nanofiber and an external magnetic field implied in increased killing of A549 cancer cell	Samadi et al., 2018 [413]
CS/polyacrylic acid/Fe_3_O_4_ magnetic nanocomposite hydrogel	Potential anticancer drug delivery system of 5-fluorouracil (5-FU)	The drug release tests showed that the CS/PAA/Fe_3_O_4_ enhanced the stability of drug dosing for a long time with controlled releases in the colon and rectal conditions, indicating a potential use of this material in DDS as a colon and rectal administration of 5-FU	Amini-Fazl et al., 2019 [426]
CS–Alg hydrogel encapsulated with magnetic gelatin microspheres (MGM)	Injectable hydrogel scaffolds for anti-cancer drug delivery of 5-fluorouracil (5-FU)	With a 30 mg/mL concentration of MGMs, the composite hydrogel provided a suitable performance and showed excellent self-healing ability under physiological conditions. Moreover, this composite hydrogel showed the sustained in vitro 5-FU release compared with control MGMs and carboxyethyl chitosan (CEC)-oxidized alginate (OAlg) hydrogel. So, this magnetic gel scaffold can render the formulation of a therapeutically effective platform for cancer treatment	Chen et al., 2019 [427]
CMCS-based nanoparticles (AM NPs) with N-(3-Aminopropyl)-imidazole (AM NPs) and perfluorobutyric anhydride (fluorinated nanoparticles -FM NPs)	pH-sensitive NPs for the delivery of Doxorubicin (DOX)	The concentration of DOX in blood for AM or FM NPs formulations was higher than that of free DOX, and it is also observed a decline of drug level in plasma with a prolonged time. In vivo DOX distribution and antitumor experiment further verified that surface-fluorinated NPs could deliver more DOX into tumor area than non-modified NPs, leading to the best tumor suppression. CMCS-based nano-carriers could prolong the blood circulation time of DOX and improve the bio-availability of the drug in vivo	Cheng et al., 2019 [428]
Norcantharidin-conjugated carboxymethyl chitosan (CMCS-NCTD)	Biopolymer carrier for a useful anti-tumor compound with severe nephrotoxicity	CMCS-NCTD could significantly inhibit tumor cells’ migration and displayed excellent anti-metastasis effects both in vitro and in vivo in a dose-dependent manner (*P* < 0.05). The enhanced antitumor effects of CMCS-NCTD were confirmed by inhibiting the growth of solid tumors and extending the survival time of tumor-bearing mice. CMCS-NCTD could inhibit tumor angiogenesis and reduce degradation of the extracellular matrix by regulating the expressions of VEGF, MMP-9, and TIMP-1	Chi et al., 2019 [429]
CS-GCNCs loaded with 5-FU	Photothermal cancer therapy (PTT)	After coating with CS, there was a significant reduction in the cytotoxicity of GCNCs, cell cycle defects, and in the 5-FU release rate of CS-GCNCs, implying in a sustained release, which could be up by 808 nm laser and microwave co-irradiation. Additionally, it was observed an enhance in the level of caspase-3 expression due to the high retention in cancer cell killing bioactivity by the 5-FU in CS-GCNCs, which implies in a higher apoptosis-related cysteine peptidase	Guo et al., 2019 [407]
Light-cured glycol chitosan (GC) hydrogel containing paclitaxel (PTX)-complexed beta-cyclodextrin (β-CD) (GC/CD/PTX)	Ovarian cancer (OC) therapy	The tumor volumes in the control and free PTX-treated samples increased gradually, whereas GC/PTX- and GC/CD/PTX-treated samples had a gradual decrease for 7 days, resulting in overall necrosis tumor tissue. A single local administration of GC/PTX showed a superior antitumor effect in the OC mouse model than that of systemic administration of free PTX solution via intravenous injection	Hyun et al., 2019 [430]
Methyl methacrylate (MMA) modified chitosan (CS) conjugate (CSMMA)	Potential gene and drug delivery agent of Curcumin	In vitro drug release study of curcumin loaded CSMMA nanoparticles demonstrated an initial burst release due to the drug entrapped near the surface of NPs, followed by a controlled release of curcumin. Additionally, pH 5.0 was more favorable than the physiological pH for the drug release. CSMMA proved to be a reasonably good gene delivery agent based on transfection efficiency studies in mammalian cancer cell lines (A549, HeLa, and HepG2)	Jaiswal et al., 2019 [431]
CS/PLA/5-FU/g-C3N4-DOX/g-C3N4-PTX triaxial nanofibers	Simultaneous controlled release of 5- fluorouracil (5-FU), Doxorubicin (DOX), and Paclitaxel (PTX) for breast cancer treatment	The maximum MCF-7 breast cancer cells killing was found to be 94.2% using tri-layer nanofibers containing intermediate and shell flow rates of 1 mL/h and 0.5 mL/h, respectively after 14 days incubation time	Jouybari et al., 2019 [432]
Hybrid lipid-CS NPs loaded with cisplatin	A platform for the controlled release of cisplatin in cancer therapy	It was confirmed the cytotoxic effect on the A2780 ovarian cancer cell line over 48 h and an increase in cellular uptake of LPHNPs. Controlled release behavior of cisplatin with a longer average residence time and half-life was evidenced, and toxicity studies in rats supplied a safety profile of LPHNPs	Khan et al., 2019 [283]
CS nanogels	Nanocarriers of polyoxometalates (POM) for breast cancer therapies	The nanocomposites have demonstrated a pH-responsive release capability of POMs, which lets the delivery of polyanions at acidic pH. So, CS nanocarriers would present significant potential for POM delivery into tumoral cells due to their pH-triggered deliverability that inhibits cytotoxic drug release at physiological pH	Pérez-Álvarez et al., 2019 [433]
PEO-CS nanofibers containing carboxymethyl-hexanoyl CS/ dodecyl sulfate NPs loaded with pyrazoline H3TM04	Skin cancer treatment	When utilizing PEO-CS nanofibers as carriers, it was observed an increased transport rate of H3TM04 through the epidermis. In in vitro cytotoxicity assays, the incorporation of H3TM04 into the nanocarriers increased its cytotoxic effect toward B16F10 melanoma cells	Rengifo et al., 2019 [434]
Fe_3_O_4_ @chitosan/Tragacanth Gum nanocomposite	Nanocomposite for Curcumin delivery	The release behavior of curcumin was studied in two different pH 7.4 and 3.4 and 37 °C and 40 °C. The nanocomposite showed a higher swelling ratio at pH 3.4 and temperature 40 °C with suitable pH and thermosensitive in vitro drug release profiles, indicating a good potential for delivery of chemotherapeutic drugs	Shafiee et al., 2019 [435]
Bio-nanocomposite beads of ZnO/CMC/CS	Environmentally friendly intelligent vehicles for colon-specific drug delivery	This nanocomposite was effectively loaded with 5-FU and showed pH-sensitivity, biodegradation capacity, and self-sustaining release behavior, relying on the content of CMC, CS, and ZnO nanoparticles	Sun et al., 2019 [299]
Ellagic acid (EA) encapsulated in CS and coated by eudragit S100 (ES100) microparticles	Improve the target and cytotoxic effect of EA on colon cancer cells	Results indicated that the generated material exhibits improved colon targeting and enhanced cytotoxic and proapoptotic activity against HCT 116 colon cancer when compared with the administration of raw EA	Alhakamy et al., 2020 [280]
